# A truncated and catalytically inactive isoform of KDM5B histone demethylase accumulates in breast cancer cells and regulates H3K4 tri-methylation and gene expression

**DOI:** 10.1038/s41417-022-00584-w

**Published:** 2023-01-26

**Authors:** Elena Di Nisio, Valerio Licursi, Cecilia Mannironi, Valentina Buglioni, Alessandro Paiardini, Giulia Robusti, Roberta Noberini, Tiziana Bonaldi, Rodolfo Negri

**Affiliations:** 1 Department of Biology and Biotechnologies “C. Darwin”, Sapienza University of Rome, via dei Sardi 70, 00185 Rome, Italy; 2 MRC Protein Phosphorylation and Ubiquitylation Unit, School of Life Sciences, University of Dundee, Sir James Black Centre, Dow Street, DD1 5EH Dundee, Scotland UK; 3 Institute of Molecular Biology and Pathology (IBPM), National Research Council (CNR) of Italy, Via degli Apuli 4, 00185 Rome, Italy; 4 Department of Biochemical Sciences, Sapienza University of Rome, p.le Aldo Moro 5, 00185 Rome, Italy; 5 Department of Experimental Oncology, IEO, European Institute of Oncology IRCCS, Via Adamello 16, 20139 Milan, Italy; 6 Department of Oncology and Hematology-Oncology, University of Milan, Milan, 20122 Italy

**Keywords:** Breast cancer, Gene expression analysis, Cell biology

## Abstract

KDM5B histone demethylase is overexpressed in many cancers and plays an ambivalent role in oncogenesis, depending on the specific context. This ambivalence could be explained by the expression of KDM5B protein isoforms with diverse functional roles, which could be present at different levels in various cancer cell lines. We show here that one of these isoforms, namely KDM5B-NTT, accumulates in breast cancer cell lines due to remarkable protein stability relative to the canonical PLU-1 isoform, which shows a much faster turnover. This isoform is the truncated and catalytically inactive product of an mRNA with a transcription start site downstream of the PLU-1 isoform, and the consequent usage of an alternative ATG for translation initiation. It also differs from the PLU-1 transcript in the inclusion of an additional exon (exon-6), previously attributed to other putative isoforms. Overexpression of this isoform in MCF7 cells leads to an increase in bulk H3K4 methylation and induces derepression of a gene cluster, including the tumor suppressor *Cav1* and several genes involved in the interferon-alpha and -gamma response. We discuss the relevance of this finding considering the hypothesis that KDM5B may possess regulatory roles independent of its catalytic activity.

## Introduction

Histone methylation is a dynamic modification that is tightly controlled during cellular differentiation by the coordinated key action of histone methyltransferases and demethylases [1]. Histone lysine demethylases (KDMs) act as both readers and erasers of this modification. Specifically, there are two enzymatic classes of histone demethylases in human: the FAD-dependent amine oxidases, belonging to the lysine-specific histone demethylase (LSD) family, also known as the KDM1 subfamily, and the Fe(II) and 2-oxoglutarate-dependent oxygenases with a conserved JmjC catalytic domain, belonging to the Jumonji histone demethylases (JHDMs) family, also known as the KDM2-KDM7 subfamilies [2, 3]. KDMs show a high level of substrate specificity, with each acting on specific lysine residues with different degrees of methylation. Histone methylation may lead to different transcriptional outcomes, such as gene activation or repression, depending on the residue involved, the degree of methylation, and the location of the mark in the genome [4, 5]. Among the KDMs, KDM5 (or JARID1) enzymes act on H3K4me2/me3, playing an important role in transcriptional regulation, especially during development and differentiation. KDM5 enzymes can act as epigenetic repressors by removing the transcriptional activating mark H3K4me3 at gene promoter regions, but they can also act as transcriptional activators by removing H3K4me3 from the body of actively transcribed genes to safeguard transcriptional elongation by repressing spurious intragenic transcription [6]. Moreover, they can contribute to transcriptional activation by converting H3K4me3 to H3K4me1 at enhancer regions, as H3K4me1 modification combined with acetylated H3K27 is predictive of active enhancers. KDM5 proteins are also involved in DNA double-strand break repair [7, 8] and chemoresistance [9–11]. The mammalian KDM5 subfamily consists of KDM5A (also known as JARID1A or RBP2), KDM5B (also known as JARID1B or PLU-1), KDM5C (also known as JARID1C or SMCX), and KDM5D (also known as JARID1D or SMCY). The deregulated expression of KDM5 enzymes has been extensively documented in many cancer types and contributes significantly to tumor initiation and progression [12]. Specifically, KDM5B is a master regulator of H3K4-methylome in stem cells, development, and cancer [13]. It was initially identified as a markedly upregulated gene in breast cancer [14] and was later found to be overexpressed in prostate [15], bladder, and lung cancers [16], and in stem-like subpopulations in melanomas [17]. In normal adult tissues, KDM5B shows a highly restricted expression, except in the testis, where it may contribute to transcriptional control during spermatogenesis, suggesting that it could belong to the class of testis/cancer antigens [18]. In breast cancer and breast cancer cell lines, KDM5B shows copy number gain associated with increased transcript levels, especially in luminal breast cancer subtypes. However, it has been suggested that KDM5B may have different functions in luminal and basal breast cancer cell lines. Indeed, analysis of differentially expressed genes following KDM5B depletion showed that in luminal cells, more genes were upregulated than downregulated, whereas the opposite was observed in basal-like cells [19]. Interestingly, luminal and HER2-positive breast cancer cell lines are particularly responsive to KDM5 catalytical inhibition or silencing [9, 11, 19–21], whereas basal lines are not [22, 23]. In the MCF7 luminal estrogen-responsive breast cancer cell line, KDM5B is involved in the repression of several tumor suppressor genes, such as BRCA1 and CAV1 [24]. In contrast, in the triple-negative breast cancer cell line MDA-MB-231, KDM5B has been shown to repress genes involved in cell proliferation, inflammatory response, adhesion and migration through interaction with the LSD1/NuRD complex, and inhibition of its expression appears to stimulate these functions [19, 23, 25, 26]. This tumor suppressor role seems specific for KDM5B, since selective inhibition of KDM5A in the same cells causes upregulation of p27 and growth arrest at the G1 phase [27]. Several transcripts are generated from the human KDM5B gene, which is expressed at different levels in normal and cancerous tissues. Recently, it has been proposed that the relative abundance of different KDM5B isoforms may contribute to tumor progression in melanoma [28, 29]. Indeed, the expression of KDM5B was detected using two types of primary antibodies specific to either the total pool of proteins or only to its RBP2-H1 isoform, resulting from a splicing variant that includes an alternative exon (exon-6) [30]. These results indicated that malignant transformation of melanocytes is not associated with the enhancement of total KDM5B expression, but rather with a change in the relative expression of its isoforms. In particular, a significant increase in RBP2-H1 expression was observed in melanomas compared to nevi. Moreover, the expression level of the RBP2-H1 variant is associated with poor prognosis in melanoma [28]. Here, we describe a previously predicted, but never experimentally characterized, KDM5B isoform that is differentially expressed in different cancer cell lines. It is a truncated and catalytically inactive isoform of KDM5B (hereafter referred to as KDM5B-NTT for N-Terminal Truncated), generated by an mRNA with a transcription start site located downstream to that described for PLU-1. This mRNA skips the first translation start site in exon-1 and instead uses a downstream ATG in exon-4. KDM5B-NTT also differs from the canonical PLU-1 isoform in the inclusion of 36 residues encoded by the variant exon-6, previously attributed to other putative isoforms. We showed that overexpression of this catalytically inactive isoform in MCF7 cells leads to an increase in bulk H3K4 methylation and induces significant transcriptomic changes. We discuss the relevance of these findings considering the hypothesis that KDM5B may possess regulatory roles independent of its catalytic activity.

## Materials and methods

### Cell cultures and extracts

The human breast cancer cell lines MCF7, T47D, and MDA-MB-231, and the human melanoma SK-MEL-28 cell line were a gift from Dr. Lucia Gabriele (Istituto Superiore di Sanità, Rome, Italy). Cells were cultured at 37 °C in a 5% CO_2_ atmosphere for no more than 10–12 in vitro passages. The DU4475 cells and other T47D cells were obtained from the MRC-PPU, University of Dundee (Scotland, UK). The MCF10A, MDA-MB-415, MDA-MB-453, SKBR3, MDA-MB-436, MDA-MB-468, SUM-149-PT, and BT-549 were obtained from the European Institute of Oncology (IEO, Milan, Italy). Mycoplasma contamination was assessed monthly (Abm, US). MCF7 and MDA-MB-231 cells were grown in high-glucose DMEM with sodium pyruvate (Corning, US), containing 10% FBS (Corning, US), 1 mmol/L l-glutamine (Sigma-Aldrich, DE), 100 U/mL penicillin, and 100 µg/mL streptomycin (Sigma-Aldrich, DE). SK-MEL-28 cells were grown in RPMI 1640 medium (Corning, US) containing 10% FBS, 1 mmol/L L-glutamine, 100 U/mL penicillin, and 100 µg/mL streptomycin. T47D cells were grown in RPMI 1640 medium (Corning, US) containing 10% FBS, 1 mmol/L L-glutamine, 10 μg/mL bovine insulin (Sigma-Aldrich, DE), 100 U/mL penicillin, and 100 µg/mL streptomycin (Sigma-Aldrich, DE). DU4475 cells were grown in RPMI 1640 medium (Gibco, UK) added with 10% FBS (Gibco, UK), 1 mmol/L L-glutamine (Gibco, UK), 100 U/mL penicillin (Gibco, UK), 100 µg/mL streptomycin (Gibco, UK) and 1 mM sodium pyruvate (Gibco, UK). MCF10A were cultured in Dulbecco’s modified Eagle’s medium (DMEM) (Euroclone) and Ham’s F12 (Gibco, UK) medium (1:1 v/v) with stable glutamine, 5% Horse Serum, 0.5 mg/mL Hydrocortisone, and 100 ng/mL Cholera toxin, with the addition of 10 µg/mL EGF at each passage. SKBR3, MDA-MB-415, and MDA-MB-453 were grown in DMEM with stable glutamine supplemented with 10% North American FBS (HyClone). BT549 were grown in DMEM with stable glutamine supplemented with 10% South American FBS (Microgem). ZR-751 were grown in RPMI 1640 (Euroclone) supplemented with 10% South American FBS, 2 mM L-Glutamine, 1 mM Sodium Pyruvate, and 10 mM Hepes. MDA-MB-468 and MDA-MB-436 were cultured in DMEM and Ham’s F12 medium (1:1 v/v) with stable glutamine and supplemented with 10% South American FBS. SUM-149-PT were grown in Ham’s F12 media supplemented with 5% South American FBS, 2 mM l-Glutamine, 5 µg/mL insulin, 1 µg/mL Hydrocortisone, and 10 mM Hepes. All media were supplemented with penicillin (100 µg/mL), and streptomycin (100 mg/mL). Cells were cultured at 37 °C in a 5% CO_2_ humidified atmosphere and passaged at 80% confluence. SUM-149-PT cell line was cultured at 37 °C in a 10% CO_2_ humidified atmosphere. Epstein–Barr virus-immortalized lymphocytes and peripheral blood mononuclear cells (PBMC) were kindly provided by Dr. Alessandra Fragale (Istituto Superiore di Sanità, Rome, Italy). Human testicular total protein lysates were purchased from Takara Bio (Shiga, US).

### Protein extraction and quantification

Cell lysis was carried out in RIPA Buffer (ThermoFisher Scientific, US) in the presence of protease inhibitor cocktail (Complete, Roche, US) and phosphatase inhibitors (Cocktail Tablets, Roche, US) according to the manufacturer’s protocol guidelines. Proteins were quantified using the Pierce Detergent Compatible Bradford Assay Kit (ThermoFisher Scientific, US).

### Western blot

KDM5B protein isoforms were analyzed by western blotting. For each sample, 30 µg of total protein was run on a denaturing 5% PAGE prepared from acrylamide/bisacrylamide solution 37.5:1 (Serva, DE) to resolve the PLU-1 (175.7 kDa) and KDM5B-NTT (161.5 kDa) isoforms. Proteins were transferred to nitrocellulose membranes (Amersham Protran WB membrane; Merck, DE, USA). The transfer efficiency was verified using the Ponceau S solution (PanReac, ES, USA). Membranes were blocked in TBS containing 0.1% Tween-20 (T-TBS) (Bio-Rad, US), 5% milk (Sigma-Aldrich, DE), or 5% (bovine serum albumin BSA (Sigma-Aldrich, DE). Hybridization was performed with rabbit KDM5B C-terminus-specific antibody (Ab) (Atlas Antibodies, HPA0217179) at a 1:7000 dilution in 5% BSA T-TBS at 4 °C overnight. Restore Western Blot Stripping Buffer (ThermoFisher Scientific, US) was used according to the manufacturer’s guidelines. Stripping efficiency was analyzed by chemoluminescence with a ChemiDoc XRS (BIO-RAD) using the Clarity Western ECL Substrate Peroxide solution/Enhancer solution (BIO-RAD, Cat. No. 1705061). After stripping, membranes were hybridized with mouse KDM5B exon-6 specific Ab (BIO-RAD, MCA4340Z) at 1:1000 dilution in 5% BSA T-TBS at 4 °C overnight. The anti-Vinculin Ab (Abcam, AB129002) was used at a dilution of 1:1000. Secondary HRP peroxidase-conjugated goat anti-rabbit Ab (ThermoFisher Scientific, US) and anti-mouse antibody (Cell Signaling, US) were used at a dilution of 1:10000. Chemoluminescence was quantified using a ChemiDoc XRS + Imaging System (Bio-Rad, US). A Spectra Multicolor High Range Protein Ladder (ThermoFisher Scientific, US) and Page Ruler Protein Ladder (ThermoFisher Scientific, US) were used. Molecular weights were calculated using the MW analysis tool of Image Lab 3.0 software. For quantitative analysis, the KDM5B-NTT isoform signal was normalized to the PLU-1 signal detected on the same blot (hybridized with α-C term of KDM5B) to evaluate the percentage fraction of KDM5B-NTT over PLU-1. The other primary Abs used after nucleus-cytoplasm fractionation (ThermoFisher Scientific, US), are as follows: lamin A/C Ab (636): sc-7292 (1:1000) (Santa Cruz), α-Tubulin Ab (B-5-1-2) T5168 (1:4000) (Sigma-Aldrich), GAPDH (Glyceraldehyde-3-phosphate dehydrogenase) Ab (FL-335): sc-25778 (1:5000) (Santa Cruz).

### 5′RLM-RACE

The 5′RACE assay was performed using the FirstChoice RLM-RACE Kit (ThermoFisher Scientific, US). RNA ligase-mediated rapid amplification of cDNA ends (RLM-RACE) was designed to amplify cDNA only from full-length capped mRNA. Briefly, total RNA from MCF7 cells was treated with CIP phosphatase to dephosphorylate uncapped RNAs and then de-capped using the TAP enzyme. After the de-capping reaction, an RNA adapter oligonucleotide was ligated to the residual phosphorylated 5′-end from the capped-mRNAs using T4 RNA ligase. RNA quality was checked after each enzymatic reaction, before RT. After a random-primed RT reaction, allowing the production of a cDNA population encompassing the 5′- initiation sites fused to the anchor, two nested PCR amplified the 5′-end of a specific transcript. As a negative control, total dephosphorylated RNA, untreated with TAP de-capping enzyme (negative TAP-control), was used in the PCR reactions. Both nested PCRs gave rise to a dominant product, which was purified and sequenced, whereas non-specific products were obtained using a Minus-TAP control reaction as a template (data not shown). Platinum Taq DNA Polymerase, High Fidelity (ThermoFisher Scientific, US) was used for PCR reactions. The primer sequences used for the 5′RACE PCR reactions are listed in Supplementary Table 1. In the first 5′RACE PCR, a Reverse Ex-6 primer and the 5′RACE Outer Fw primer were used; in the second nested 5′RACE PCR, the downstream 5′RACE Inner Fw primer was used together with the reverse primer 1 (PCR1) or the reverse primer 2 (PCR2); both Fw primers were complementary to the 5′-anchor sequence and provided with the kit. The images were acquired using a Typhoon 9300 scanning instrument. The purified PCR products were analyzed by Sanger sequencing.

### Statistical analysis

Data were analyzed using R version 4.2, Prism (version 6.0; GraphPad Software Inc.) and Microsoft Excel version 16. Statistical analysis was conducted using unpaired two-sided Welch’s *t*-test when the variances of experimental groups were not similar, or one-way ANOVA for normal distributed values, as indicated in the figure legends. RNA-Seq statistical analysis information are reported in Supplementary Information file. *p*-value of less than 0.05 was considered statistically significant. Error bars reported in graphs as S.D. Sample size (n) values are indicated on the corresponding graphs or in the legend. All replicates in every experiment were collected independently.

## Results

### Characterization of a truncated KDM5B isoform

**Fig. 1:**
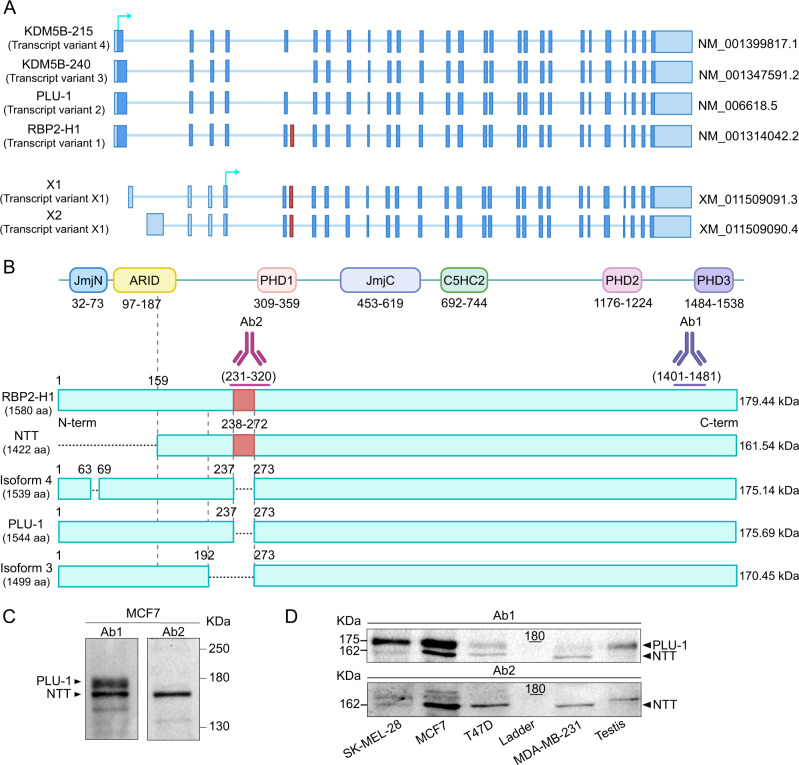
KDM5B transcripts and protein isoforms. **A** KDM5B transcripts as reported in Ref Seq NCBI database. RBP2-H1, PLU-1, KDM5B-240, and KDM5B-215 are the validated (NM) variants, X1 and X2 are the predicted (XM) ones. Transcripts differ at the 5′ UTRs and for the presence of alternative exons. RBP2-H1 represents the longest transcript and includes the exon-6 (in red). The arrows indicate the ATG sites, the first one in exon-1 and the second one in exon-4. **B** Protein domains of human KDM5B are reported above validated and predicted KDM5B protein isoforms; PLU-1 is the canonical isoform. Ab1 and Ab2 are KDM5B C-terminus and exon-6 specific antibodies, respectively. **C** WB analysis of KDM5B isoform expression in MCF7 lysates: on the left two bands corresponding to 175.7 and 161.5 kDa proteins are detected by Ab1 in the first blot, while on the right one single band for 161.5 kDa protein is detected by Ab2, after the stripping of the first blotting. **D** Preliminary investigation of KDM5B isoforms’ expression in different human cell lines and human testis, using the two antibodies Ab1 (panel up) and Ab2 (panel down), before and after the stripping, respectively. Panels A and B created with BioRender.com.

**Fig. 2:**
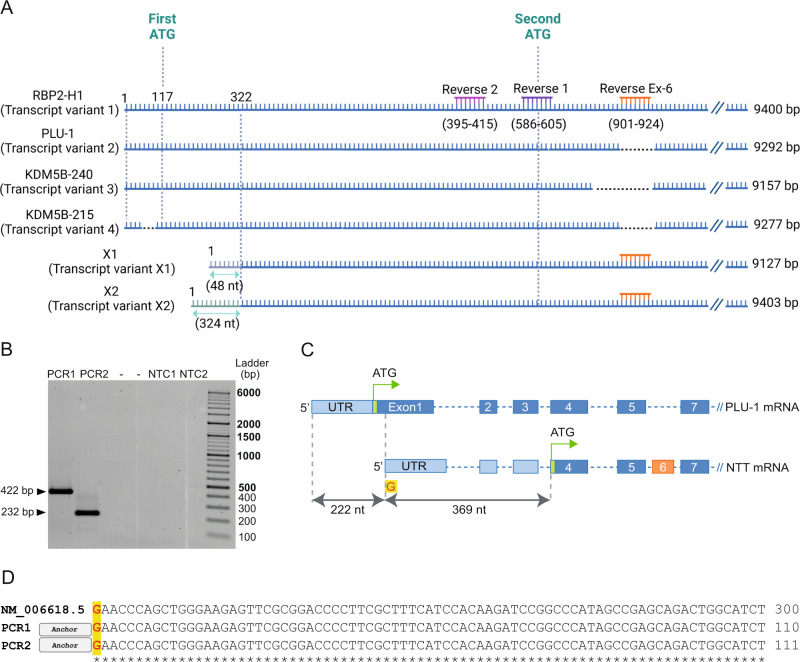
5′RLM-RACE analysis of KDM5B transcripts. **A** The scheme reports the position of reverse primers used for the two RT-PCRs of 5′RACE experiment. Binding nucleotides are indicated underneath RBP2H1 sequence. See Material and Methods paragraph for experimental details. **B** PCR products from PCR1 and PCR2. Product lengths are as expected. **C** The position of first and second ATG codons are shown with green arrows. We found a new 5′-end 221 nucleotides downstream the canonical one. **D** Partial alignment of PCR1 and PCR2 sequences refer to KMD5B-PLU1 transcript. (For the complete alignment see Supplementary File S1). Panel A created with BioRender.com.

The NCBI Reference Sequence (RefSeq) database retrieved four validated (NM) transcripts of KDM5B: PLU-1, KDM5B-215, KDM5B-240, and RBP2-H1 mRNAs. As shown in Fig. 1A, they differ in the inclusion of exon-5 and exon-6. Specifically, RBP2-H1 represents the longest transcript and includes exon-6 that was absent in KDM5B-215, KDM5B-240, and PLU-1. KDM5B-240 lacks exon-5 and KDM5B-215 uses an alternative splice site in exon-1 compared to the other transcripts. The RBP2-H1 protein contains 36 additional residues after residue 237 of the original PLU-1 sequence, with a predicted molecular weight of 179 kDa. Nevertheless, some authors attribute this to the apparent molecular weight of 160 kDa [31]. Exon-6 sequence has strong homology with Alu sequences; however, its functional role in the KDM5B context is still unknown [18]. KDM5B protein isoforms generated from PLU-1, KDM5B-215 and KDM5B-240 transcripts have a molecular weight of 175.7 kDa, 175.1 and 170.5 kDa, respectively. Interestingly, in the NCBI RefSeq Database, two additional transcripts of KDM5B were annotated: X1 and X2 [32]. The exon-1 of the two predicted transcripts falls within the intronic region of the validated transcripts. Both X1 and X2 transcripts include exon-6 and differ from each other only in the 5′UTR region. X1 and X2 transcripts encode for a never-validated protein isoform, namely the X1 protein. These transcripts utilize an alternative transcription start site located downstream of the canonical, including exon-6 and are translated starting from an ATG located downstream to the canonical one (Fig. 1A). Indeed, the translation of these two predicted transcripts would not start from the first exon but from the fourth exon, thus producing proteins smaller than the other previously described isoforms. Since the only difference between the X1 and X2 transcripts lies in the 5′-UTR, both would give rise to the same protein isoform which, compared to PLU-1, would have a truncation of 158 residues in the N-terminal region, but would gain 36 additional residues corresponding to exon-6. Consequently, the molecular weight of this predicted protein isoform was 161.5 kDa. To distinguish this putative isoform from the longer ones, we ran total protein extracts from breast cancer cell lines in high-resolution SDS-PAGE, focusing on the 130–180 kDa protein MW range. The gels were subjected to western blotting and hybridized with two different antibodies, one recognizing a carboxy-terminal antigen common to all the predicted isoforms (Ab1, see Materials and Methods) and the other specific for exon-6-encoded residues (Ab2) (Fig. 1B). As shown in Fig. 1C, in a total extract from the MCF7 breast cancer cell line, Ab1 recognized two major bands with apparent molecular weights of approximately 176 and 162 kDa, consistent with the presence of PLU-1, and a smaller protein that might be produced by X1 or X2 transcripts. A second hybridization with Ab2 showed that exon-6 was almost exclusively contained in the 162 kDa band, confirming its hypothetical origin. Figure 1D shows that the 162 kDa band is also evident in other cancer cell lines, such as breast cancer T47D and MDA-MB-231 and melanoma SK-MEL-28, whereas it is barely detectable in a cell extract from the testis. Once again, hybridization with Ab2 showed that exon-6 is mostly present in the shorter isoform. This result suggests that a truncated KDM5B isoform (KDM5B-NTT) produced by an alternative mRNA, initiated downstream of the canonical ATG, exists and is strongly expressed in different cancer cell lines. To validate this hypothesis, we set up a 5′-RACE mapping experiment aimed at identifying a capped mRNA downstream of the canonical ATG and including exon-6 (Fig. 2A). The 5′RACE allowed precise mapping of the transcriptional start in the region between the first and second ATG, specifically at 369 nucleotides upstream of the second translation start site (Fig. 2B and C). Figure 2D shows that the sequences found with 5′RACE were identical to those of PLU-1 (the complete sequences and alignments are shown in Supplementary File S1).

**Fig. 3:**
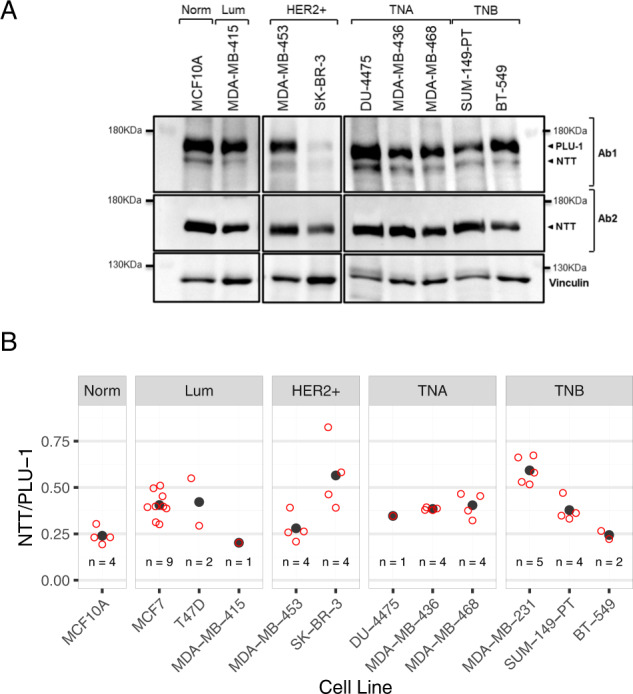
Expression of KDM5B isoforms in different subtypes of breast cancer cell lines. **A** WB analysis of KDM5B isoforms’ expression in lysates of different breast cancer cell lines shows that NTT isoform is detected in all of them; as confirmed also by the blotting using the Ab2, the exon-6-encoded residues are almost exclusively included in the truncated isoform. Cell lines are grouped according to the molecular subtypes. **B** No correlation between breast cancer subtypes and relative NTT expression (calculated as NTT/PLU-1 ratio) is highlighted upon the quantification.

The preliminary analysis presented in Fig. 1 suggests that KDM5B-NTT could be differentially expressed in different cancer cell lines. We focused on a panel of breast cancer cell lines classified as luminal, HER2+, triple-negative A, and triple-negative B and tested the expression of the truncated isoform in the different subtypes. Figure 3A shows that KDM5B-NTT is present in all breast cancer cell lines. In all cases, hybridization with Ab2 confirms that exon-6 is almost exclusively included in the truncated isoform. Quantitative analysis shows that the KDM5B-NTT/PLU-1 ratio varies between 0.2 and 0.7 without a clear correlation with the cancer subtype classification (Fig. 3B). In particular, the KDM5B-NTT isoform appeared to be relatively more highly expressed in MDA-MB-231 basal breast cancer cell lines than in MCF7 luminal breast cancer cell lines (Figs. 1D, 3B, and S1). This observation is interesting because of the different functional roles of KDM5B in the MDA-MB-231 cell line, where it inhibits growth and metastatic capability [22], and in MCF7 cell line, where it sustains proliferation. Our quantification (Fig. S1) confirms previous observations, indicating that global KDM5B expression is significantly higher in MCF7 than in MDA-MB-231 cells [20, 23]. Interestingly, our analysis clearly indicated that the NTT/PLU-1 ratio is significantly higher in MDA-MB-231 (0.57 average) than in MCF7 cells (0.40 average), (Fig. 3B and S1A–C). Next, we investigated whether this difference was due to transcriptional regulation. Therefore, by RT-qPCR we measured the amount of exon-6 containing mRNA relative to the total KDM5B mRNA in the two cell lines. As shown in Fig. S1E, the fraction level of exon-6 containing mRNA, representing KDM5B-NTT mRNA, was very similar in the two cell lines accounting for a very low portion of the total KDM5B mRNA (approximately 4–9%), although the global level of KDM5B transcripts was considerably higher in MCF7 cells, as previously observed (Fig. S1D). Semi-quantitative RT-PCR also showed that in T47D and SK-MEL-28 exon-6 containing mRNA was very limited and similar to that detected in MCF7 and MDA-MB-231 cells, whereas it was barely detectable in human PMBC or EBN-immortalized lymphocytes (Fig. S2).

### KDM5B-NTT is mainly regulated at the protein level

**Fig. 4:**
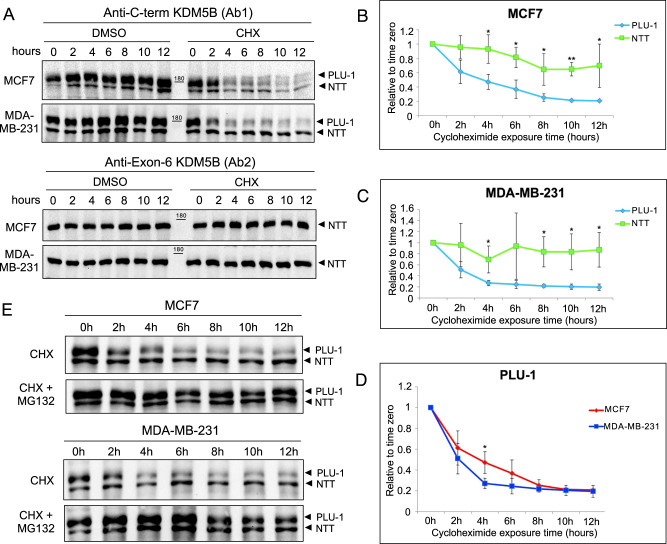
Protein stability of KDM5B isoforms. **A** After CHX treatment, the KDM5B-NTT results more stable than KDM5B-PLU-1 in both MCF7 and MDA-MB-231, as shown from the blots using the anti-C-terminal KDM5B (upper panel) and the anti-exon-6 KDM5B (lower panel) antibodies. **B** Degradation kinetics of KDM5B isoforms in a time course in MCF7 cells (*n* = 3 for each time point). **C** Degradation kinetics of KDM5B isoforms in a time course in MDA-MB-231 cells (*n* = 3 for each time point). **D** Comparing the degradation curves of PLU-1 isoform in the two analyzed breast cancer cell lines, it was found a different rate of degradation, that is significantly faster in MDA-MB-231, where the mean half-life-time of the PLU-1 is of about 2 h, versus MCF7, where instead it is around 4 h (*n* = 3 for each time point). **E** The cycloheximide (CHX) treatment highlights the PLU-1 instability, which is rescued by MG132 treatment, demonstrating that the proteasomal activity regulates the turnover of PLU-1 isoform in MCF7 and MDA-MB-231. ns: *p* > 0.05, *p < = 0.05, **p < = 0.01, ***p < = 0.001; Statistics by unpaired Welch’s *t*-test.

Our analysis raises the question of how transcript, representing less than 10% of the total KDM5B mRNA, can generate a much higher percental fraction of the total KDM5B protein. We envisaged two possible explanations for this: the truncated protein is either more efficiently translated or more stable. We tested the second possibility by blocking protein synthesis with cycloheximide (CHX) in MCF7 and MDA-MB-231 cell lines and monitoring the time course of degradation of the two protein isoforms. As shown in Fig. 4A–C, after 12 h of CHX treatment, the PLU1 level was less than 20% compared to DMSO-treated control cells. Instead, the residual KDM5B-NTT protein level after CHX treatment accounted for more than 60% in MCF7 cells and more than 80% in MDA-MB-231 cells, compared to DMSO-treated control cells. Our results indicate that there is a striking difference in turnover between the two isoforms: PLU-1 shows a fast turnover (T50 around 3 h), whereas KDM5B-NTT remains remarkably stable for at least 12 h. Moreover, PLU-1 turnover was faster in MDA-MB-231 cells than in MCF7 cells (Fig. 4D). This might explain the higher relative abundance of KDM5B-NTT in these cells. We also investigated whether PLU-1 turnover was related to proteasomal degradation. Figure 4E shows that proteasome inhibition by MG132 considerably slowed down protein degradation following cycloheximide block.

### KDM5B-NTT isoform affects gene expression and H3K4me3 level

**Fig. 5:**
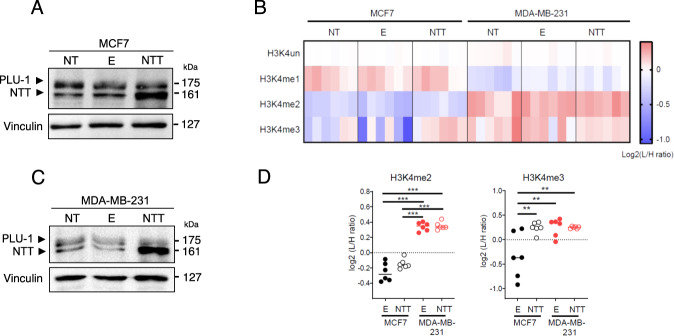
MS-based analysis of H3K4 methylation levels in MCF7 and MDA-MB-231 cells overexpressing the KDM5B-NTT isoform. **A** The KDM5B-NTT expression in the overexpression condition in MCF7 cells at 24 h from transfection increases compared to cells transfected with the Empty vector (E), where the KDM5B-NTT expression is similar to that observed in the non-transfected cells (NT). **B** The KDM5B-NTT expression increases also in MDA-MB-231 cells at 24 h from transfection in the overexpression condition compared to negative controls. **C** Heatmap display of the log2 L/H ratios (where L: samples and H: internal standard) obtained for H3K4 methylations in MCF7 and MDA-MB-231 cells not transfected (NT), transfected with empty vector (E), or overexpressing KDM5B-NTT. The data were normalized over the average value across all the samples. **D** H3K4me2 and H3K4me3 levels (expressed as L/H ratios) in MCF7 and MDA-MB-231 cells overexpressing KDM5B-NTT, compared to control E. Statistical analysis was performed by repeated measures ANOVA, followed by Tukey’s multiple comparison test. **p < 0.005, ***p < 0.001.

**Fig. 6:**
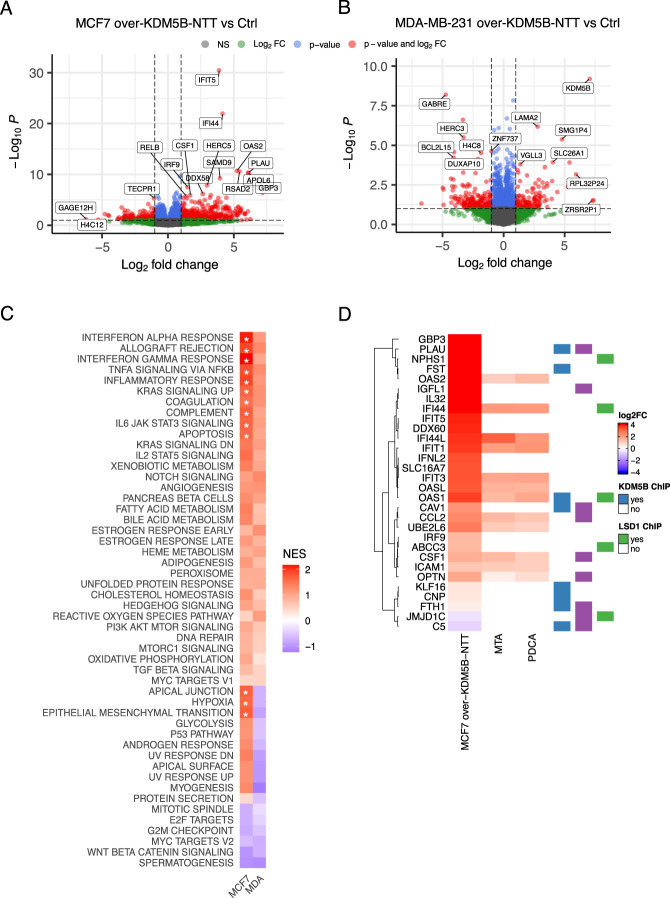
Effects of KDM5B-NTT overexpression on transcriptome. **A**, **B** Volcano plots showing the differentially expressed genes (DEGs) in MCF7 and MDA-MB-231 cells with overexpression of KDM5B-NTT, respectively. Red dots indicate DEGs associated with *p* < 0.1 and log_2_(FC) < −1 or log_2_(FC) > 1; blue dots indicate genes with *p* < 0.1 and −1 < log_2_(FC) < 1; green dots indicate genes with log_2_(FC) < −1 or log_2_(FC) > 1, and *p* > 0.1. **C** Heatmap showing in color scale the GSEA Normalized Enriched Score (NES) of gene expression profile resulting from overexpression of KDM5B-NTT in MCF7 and MDA-MB-231 cells in comparison with control cell samples. (White * = FDR < 0.01). **D** Cluster of KDM5B-NTT-deregulated genes, which are also significantly induced by one or both epigenetic drug (PDCA and MTA) and/or previously shown to be bound by KDM5B in ChIP experiments [19, 22, 25].

As KDM5B-NTT is relatively abundant in different cancer cell lines, it is important to understand its effects on cell function. Figure 1B shows a comparison of the domain compositions of PLU-1 and KDM5B-NTT. The truncated isoform lacks JmjN and a part of the ARID domains. According to previous observations, the protein should be catalytically inactive [15, 25, 33] and unable to bind to DNA through the ARID domain. However, it should retain the ability to enter the nucleus and associate with chromatin through the PHD domains or by interacting with PLU-1 regulatory partners. KDM5B-NTT indeed retained all the nuclear localization signals (Fig. S3A). We analyzed the localization of PLU-1 and KDM5B-NTT in the nuclear and cytoplasmic fractions of MDA-MB-231 cells (Fig. S3B, C). Both PLU-1 and KDM5B-NTT isoforms were highly enriched in the nuclear fraction. Next, we tested the effect of KDM5B-NTT overexpression on histone methylation. To transiently overexpress this isoform, MCF7 and MDA-MB-231 cells were transfected (Fig. S4). The protein was efficiently expressed in both cell lines, as shown by the increased signal of the 162 kDa band in the cells 24 or 48 hours after transfection (Fig. 5A, C and S4B, C). At 24 h post-transfection, the KDM5B-NTT protein was increased by 2–3-fold in MCF7 and–4-8-fold in MDA-MB-231 cells compared to the control empty plasmid (E). In the transfected cells KDM5B-NTT became more prevalent, being approximately 2- or 5-fold more abundant than PLU-1 in MCF7 and MDA-MB-231 cells, respectively (Fig. S4B, C). KDM5B-NTT overexpression in MCF7 does not show significant effects on proliferation nor in cell cycle dynamics in MCF7 cells (Fig. S5). MS-based quantification of H3K4 methylation showed higher levels of H3K4me2 and H3K4me3 in MDA-MB-231 cells than in MCF7 cells under basal conditions (Fig. 5B, D). In MCF7 cells, 24 hours after transfection with the construct for KDM5B-NTT, the level of H3K4me3 was significantly increased, reaching the level quantified in MDA-MB-231, where H3K4me3 was not significantly affected by KDM5B-NTT overexpression (Fig. 5B, D). No significant effect was observed for H3K4me2 or H3K4me1 in either cell lines (Fig. 5B, D). The increase in H3K4me3 level observed in MCF7 cells upon KDM5B-NTT overexpression can be explained by a competition effect exerted by the catalytically inactive protein on PLU-1 and eventually other KDM5 histone demethylases. This effect was not observed in MDA-MB-231 cells, which have significantly higher basal constitutive levels of H3K4me3, likely due to lower PLU-1 abundance. Moreover, the increase in H3K4me3 appeared to be lost in MCF7 cells 48 hours after transfection (data not shown) when the KDM5B-NTT protein level decreased (Fig. S4B). To evaluate the possible role of KDM5B-NTT in the overall transcription profile, we performed RNA-Seq analysis after KDM5B-NTT overexpression in MCF7 and MDA-MB-231 cells. RNA-Seq was performed on total RNA purified from four different batches of MCF7 and MDA-MB-231 cells 24 hours after transfection with the plasmid for KDM5B-NTT overexpression or empty vector. Principal component analysis (PCA), after batch effect correction, showed a clear clustering of the KDM5B-NTT transfected samples vs. empty vector in both cell lines (Fig. S4D, E). RNA-Seq data analysis showed that 123 transcripts were significantly (FDR < 0.1) modulated in MCF7 cells and only 47 in MDA-MB-231 cells. Moreover, most of the modulated genes in MCF7 cells were induced (91), and the majority (66) were induced at least 2-fold (Fig. 6A). In contrast, in MDA-MB-231, only seven genes were significantly induced and 8 repressed at least 2-fold (Fig. 6B). Gene set enrichment analysis (GSEA) showed that KDM5B-NTT overexpression led to modulation of genes belonging to the interferon-alpha and gamma response and inflammation (Fig. 6C) in MCF7 cells but not in MDA-MB-231 cells. In order to understand whether the induction in MCF7 could be due to epigenetic derepression by KDM5B-NTT, we tested the effect of two epigenetic drugs: PDCA, a selective KDM5 inhibitor [34], and MTA, a general DNA and histone methyltransferase inhibitor [35], which has been previously shown to reactivate epigenetically silenced promoters. Figure 6D shows a cluster of KDM5B-NTT-derepressed genes that were also significantly induced by one or both treatments and/or previously shown to be bound by KDM5B in ChIP experiments [19, 22, 25]. These targets are good candidates for the direct competitive derepression by KDM5B-NTT.

## Discussion

KDM5B has an ambivalent role in oncogenesis, behaving as an oncogene in some cancer cell lines and as a tumor suppressor in others [12, 36]. This could partly depend on the pattern of the isoforms expressed in different cell lines. Here, we showed that twelve different breast cancer lines express a truncated isoform in the N-terminal region, which we called KDM5B-NTT, containing exon-6, previously identified in another isoform. We showed that this protein isoform originates from a transcript starting downstream of the canonical transcription start site and uses a downstream ATG for translation. Indeed, using a bioinformatic tool for detecting active promoters based on ChIP-Seq data on transcription factor occupancy and histone modifications [19, 22] a putative active promoter can be localized downstream of the first ATG, but upstream of the second one (Fig. S6). The ratio between the canonical isoform PLU-1 and KDM5B-NTT is variable in the different cell lines without a clear correlation with the cancer subtype classification (Fig. 3B and Fig. S1). We showed that, although the downstream transcript is produced in a very low amount, the corresponding protein accumulates and is much more stable than the canonical PLU-1 isoform, which is rapidly degraded by the proteasome, as previously shown [10, 37].

RT-qPCR data (Fig. S1D, E) and the bioinformatic analysis performed with data from public repositories (CCLE and TCGA) presented in Fig. S7 suggest that the mRNA containing exon-6 is present at relatively constant and low amounts (<10%) in all cancer lines and patients samples examined, with few exceptions. The difference in expression between PLU-1 and exon-6 containing isoforms observed at the protein level in melanoma [28, 29] and breast cancer cell lines (this study) is not recapitulated at the mRNA level. This confirms that the abundance of the KDM5B-NTT isoform is mainly regulated at the post-translational level and that its increase at the protein level should be indeed due to a notable resistance of the truncated isoform to proteasome degradation.

We do not have an explanation for this remarkably different stability; however, we observed that a bona fide “Degron” protein motif [38] is present on the N-terminal portion of PLU-1 (Fig. S8) and missing in the NTT shorter isoform. Moreover, previous work provided evidence that KDM5B degradation by the proteasome could be positively regulated by SUMOylation [39], which mainly occurs at the lysine residues K242 and K278 by the action of the E3 SUMO ligase RNF4 [37]. The residue K242 is also ubiquitinated by the E3 ubiquitin ligases TRAF6 [40] and/or SKP2 [41], suggesting that KDM5B protein levels may be differentially regulated by synchronization of the ubiquitination and sumoylation machinery in cancers [40]. Strikingly, while both K242 and K248 residues map to a disordered region of PLU-1, the first one appears less accessible in the KDM5B-NTT protein at position 120, as shown in the predicted 3D protein structure (Fig. S8). Targeted experiments are needed to assess whether the N-terminal portion which is missing in KDM5B-NTT is specifically responsible to target PLU-1 to proteasomal degradation. Interestingly, the region missing in KDM5B-NTT also contains the JmjN and a portion of the ARID domains of PLU-1 and previous experiments [15, 25] have shown that constructs lacking the JmjN or ARID domains are catalytically inactive. In line with this, molecular modeling shows that a linked JmjN-JmjC domain is crucial for the efficient binding of catalytic cofactors [33, 42, 43]. We confirmed these observations by demonstrating that KDM5B-NTT overexpression did not decrease the bulk levels of H3K4 tri-methylation in vivo, which in MCF7 was instead significantly increased upon transfection. Since the protein is correctly localized to the nucleus, questions arise about its biological roles. Recently, Taylor-Papadimitru et al. [44] studied a strain of mice expressing a KDM5B splicing variant that lacked the ARID domain and five amino acids in the JmjN domain. They demonstrated that, although the protein is catalytically inactive, the mouse is viable and fertile, contrary to the KDM5B knockout mouse, which displays an embryonic lethal phenotype [45]. These data corroborate several experimental observations regarding the relevant biological effects of KDM5 demethylases that are independent of their catalytic activity [12, 37, 46–48]. Here, we hypothesized that the KDM5B-NTT protein could be recruited to genomic sites through the PHD domains and retain some non-catalytic regulatory functions of KDM5B and/or compete for the catalytic functions of the PLU-1 isoform. In this regard, the observation that the ratio between the two isoforms is significantly different between the luminal breast cancer MCF7 cell line (KDM5B-NTT around 40%) and the triple-negative/basal-like MDA-MB-231 (KDM5B-NTT around 57%) suggests a possible dominant-negative role of the inactive isoform in lowering PLU-1 activity by competing for genomic targets in the basal cell lines where high PLU-1 expression has been shown to inhibit optimal growth [23]. This hypothesis is strongly supported by our experiments, which showed that KDM5B-NTT overexpression increases H3K4me3 in MCF7 bulk chromatin to reach a higher level detected in MDA-MB-231 cells. Indeed, our transcriptomic analysis showed that KDM5B-NTT overexpression in MCF7 derepresses several genes poorly transcribed in control cells, which also respond to PDCA, a catalytic inhibitor of KDM5, and MTA, a general epigenetic derepressor (Fig. 6D). Moreover, the list of KDM5B-NTT-induced genes is highly enriched in genes belonging to interferon-alpha and gamma response and inflammation, which have a primary role in tumor progression and regression [49]. We have previously provided experimental evidence that these genes can be derepressed in colon cancer cells by epigenetic drug treatment, leading to a decrease in H3K9 methylation and a parallel increase in H3K4 tri-methylation [50]. It is conceivable that the KDM5B-NTT isoform could access some of the targets bound by KDM5B-PLU-1 and compete with its repressive activity. Indeed, a significant fraction of KDM5B-NTT-modulated genes was previously shown to bind to KDM5B in MCF7 or other breast cancer cell lines (Fig. 6). In particular, *Cav1* is considered to be a prototype of genes repressed by the direct action of PLU-1 [24, 25]. This gene, which has tumor suppressor activity in MCF7 cells [51], recruits KDM5B to the promoter. Consequently, Cav1 is H3K4me3-depleted and transcriptionally repressed [24, 25]. In contrast, KDM5B-NTT overexpression increased Cav1 expression (Fig. 6D).

**Fig. 7:**
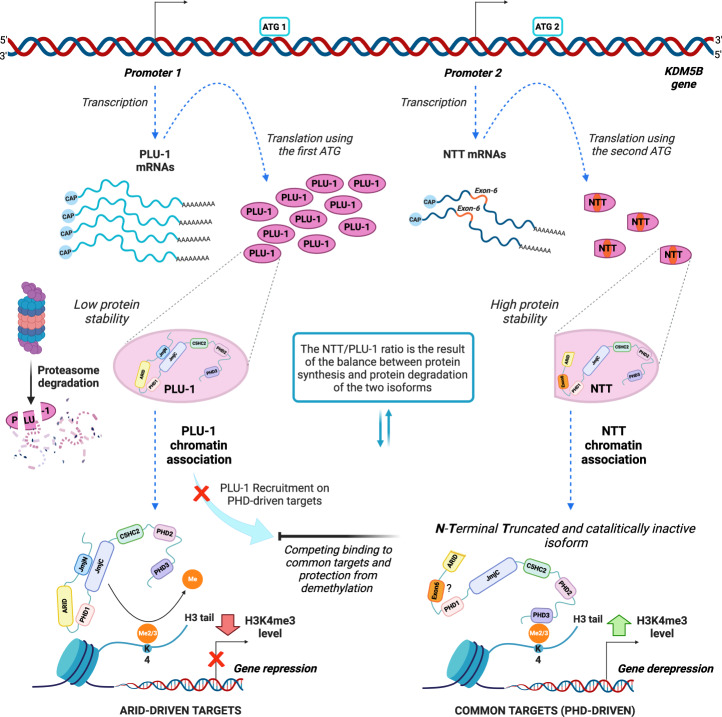
Diagram illustrating the proposed mechanism for the regulatory action of KDM5B-NTT. Transcription from Promoter 2 generates NTT mRNAs which include the exon-6, and which lead to the expression of the KDM5B-NTT protein isoform. A second ATG (ATG2) is used for translation of NTT isoform, resulting in a KDM5B N-Terminal Truncated protein isoform including 36 more residues encoded by the exon-6. Even though the NTT mRNAs are around 7% of the total KDM5B transcriptional events, the NTT protein isoform is very much more stable compared to PLU-1, targeted by the proteasome degradation system. NTT chromatin association is driven by the PHD3 domain, which preferentially recognizes the tri-methylated H3K4. This association inhibits the PLU-1 recruitment on common targets and prevents their demethylation, leading to an increase of H3K4me3 levels and resulting in gene derepression. Created with BioRender.com.

The putative dominant-negative role of KDM5B-NTT in H3K4me2/3 demethylation and transcriptional regulation is illustrated in Fig. 7.

An alternative hypothesis is that some functional effects of KDM5B-NTT could be due to the ability of the protein to locally recruit transcription and RNA-processing regulators at specific genomic binding sites, independently of its catalytic activity [22, 23, 52].

For what regards the NTT/PLU-1 ratio in the different breast cancer cell lines observed in our preliminary analysis, no clear correlation was found with the molecular subtype classification. Indeed, while the NTT expression appears quite constant in all the analyzed cell lines, the variable NTT/PLU-1 ratio is likely to be mainly affected by the balance between PLU-1 synthesis and degradation, depending on the activity of the proteasomal system in each cell line and by the physiological state of the cells. Therefore, this aspect adds a higher level of complexity, which in the future should be taken into account to fully appreciate the relevance of KDM5B-NTT in fine-tuning KDM5B-PLU-1 isoform activity in different breast cancer subtypes.

## Supplementary information

Supplementary Methods

Supplementary Tables

Supplementary File S1

Supplementary Figures

## Data Availability

The RNA-Seq accompanying this paper are available through NCBI’s Gene Expression Omnibus (GEO) repository, under accession number GSE218819 located at https://www.ncbi.nlm.nih.gov/geo/query/acc.cgi?acc=GSE218819. The mass spectrometry proteomics data have been deposited to the ProteomeXchange Consortium [53] via the PRIDE partner repository with the dataset identifier PXD033337.

## References

[1] Jambhekar A, Dhall A, Shi Y (2019). Roles and regulation of histone methylation in animal development. Nat Rev Mol Cell Biol.

[2] Klose RJ, Kallin EM, Zhang Y (2006). JmjC-domain-containing proteins and histone demethylation. Nat Rev Genet.

[3] Dimitrova E, Turberfield AH, Klose RJ (2015). Histone demethylases in chromatin biology and beyond. EMBO Rep.

[4] Bannister AJ, Kouzarides T (2011). Regulation of chromatin by histone modifications. Cell Res.

[5] Barski A, Cuddapah S, Cui K, Roh T-Y, Schones DE, Wang Z (2007). High-resolution profiling of histone methylations in the human genome. Cell.

[6] Xie L, Pelz C, Wang W, Bashar A, Varlamova O, Shadle S (2011). KDM5B regulates embryonic stem cell self-renewal and represses cryptic intragenic transcription. EMBO J.

[7] Li X, Liu L, Yang S, Song N, Zhou X, Gao J (2014). Histone demethylase KDM5B is a key regulator of genome stability. Proc Natl Acad Sci USA.

[8] Di Nisio E, Lupo G, Licursi V, Negri R (2021). The role of histone lysine methylation in the response of mammalian cells to ionizing radiation. Front Genet.

[9] Paroni G, Bolis M, Zanetti A, Ubezio P, Helin K, Staller P (2019). HER2-positive breast-cancer cell lines are sensitive to KDM5 inhibition: definition of a gene-expression model for the selection of sensitive cases. Oncogene.

[10] Xu W, Zhou B, Zhao X, Zhu L, Xu J, Jiang Z (2018). KDM5B demethylates H3K4 to recruit XRCC1 and promote chemoresistance. Int J Biol Sci.

[11] Pippa S, Mannironi C, Licursi V, Bombardi L, Colotti G, Cundari E et al. Small Molecule Inhibitors of KDM5 Histone Demethylases Increase the Radiosensitivity of Breast Cancer Cells Overexpressing JARID1B. Molecules. 2019;24. 10.3390/molecules24091739 PMC6540222 31060229

[12] Taylor-Papadimitriou J, Burchell JM (2022). Histone methylases and demethylases regulating antagonistic methyl marks: changes occurring in cancer. Cells.

[13] Xhabija B, Kidder BL (2019). KDM5B is a master regulator of the H3K4-methylome in stem cells, development and cancer. Semin Cancer Biol.

[14] Lu PJ, Sundquist K, Baeckstrom D, Poulsom R, Hanby A, Meier-Ewert S (1999). A novel gene (PLU-1) containing highly conserved putative DNA/chromatin binding motifs is specifically up-regulated in breast cancer. J Biol Chem.

[15] Xiang Y, Zhu Z, Han G, Ye X, Xu B, Peng Z (2007). JARID1B is a histone H3 lysine 4 demethylase up-regulated in prostate cancer. Proc Natl Acad Sci USA.

[16] Hayami S, Yoshimatsu M, Veerakumarasivam A, Unoki M, Iwai Y, Tsunoda T (2010). Overexpression of the JmjC histone demethylase KDM5B in human carcinogenesis: involvement in the proliferation of cancer cells through the E2F/RB pathway. Mol Cancer.

[17] Roesch A, Fukunaga-Kalabis M, Schmidt EC, Zabierowski SE, Brafford PA, Vultur A (2010). A temporarily distinct subpopulation of slow-cycling melanoma cells is required for continuous tumor growth. Cell.

[18] Barrett A, Madsen B, Copier J, Lu PJ, Cooper L, Scibetta AG (2002). PLU-1 nuclear protein, which is upregulated in breast cancer, shows restricted expression in normal human adult tissues: a new cancer/testis antigen?. Int J Cancer.

[19] Yamamoto S, Wu Z, Russnes HG, Takagi S, Peluffo G, Vaske C (2014). JARID1B is a luminal lineage-driving oncogene in breast cancer. Cancer Cell.

[20] Mocavini I, Pippa S, Licursi V, Paci P, Trisciuoglio D, Mannironi C (2019). JARID1B expression and its function in DNA damage repair are tightly regulated by miRNAs in breast cancer. Cancer Sci.

[21] Hinohara K, Wu H-J, Vigneau S, McDonald TO, Igarashi KJ, Yamamoto KN (2018). KDM5 histone demethylase activity links cellular transcriptomic heterogeneity to therapeutic resistance. Cancer Cell.

[22] Li Q, Shi L, Gui B, Yu W, Wang J, Zhang D (2011). Binding of the JmjC demethylase JARID1B to LSD1/NuRD suppresses angiogenesis and metastasis in breast cancer cells by repressing chemokine CCL14. Cancer Res.

[23] Klein BJ, Piao L, Xi Y, Rincon-Arano H, Rothbart SB, Peng D (2014). The histone-H3K4-specific demethylase KDM5B binds to its substrate and product through distinct PHD fingers. Cell Rep.

[24] Scibetta AG, Santangelo S, Coleman J, Hall D, Chaplin T, Copier J (2007). Functional analysis of the transcription repressor PLU-1/JARID1B. Mol Cell Biol.

[25] Yamane K, Tateishi K, Klose RJ, Fang J, Fabrizio LA, Erdjument-Bromage H (2007). PLU-1 is an H3K4 demethylase involved in transcriptional repression and breast cancer cell proliferation. Mol Cell.

[26] Mandumpala JJ, Baby S, Tom AA, Godugu C, Shankaraiah N (2022). Role of histone demethylases and histone methyltransferases in triple-negative breast cancer: Epigenetic mnemonics. Life Sci.

[27] Yang G-J, Wang W, Mok SWF, Wu C, Law BYK, Miao X-M (2018). Selective Inhibition of lysine-specific demethylase 5A (KDM5A) using a rhodium(III) complex for triple-negative breast cancer therapy. Angew Chem Int Ed Engl.

[28] Kuźbicki Ł, Lange D, Stanek-Widera A, Chwirot BW (2017). Prognostic significance of RBP2-H1 variant of JARID1B in melanoma. BMC Cancer.

[29] Vogt T, Kroiss M, McClelland M, Gruss C, Becker B, Bosserhoff AK (1999). Deficiency of a novel retinoblastoma binding protein 2-homolog is a consistent feature of sporadic human melanoma skin cancer. Lab Invest.

[30] Kuźbicki Ł, Lange D, Strączyńska-Niemiec A, Chwirot BW (2016). Altered splicing of JARID1B in development of human cutaneous melanoma?. Appl Immunohistochemistry Mol Morphol.

[31] Roesch A, Becker B, Meyer S, Wild P, Hafner C, Landthaler M (2005). Retinoblastoma-binding protein 2-homolog 1: a retinoblastoma-binding protein downregulated in malignant melanomas. Mod Pathol.

[32] Kapustin Y, Souvorov A, Tatusova T, Lipman D (2008). Splign: algorithms for computing spliced alignments with identification of paralogs. Biol Direct.

[33] Horton JR, Engstrom A, Zoeller EL, Liu X, Shanks JR, Zhang X (2016). Characterization of a linked Jumonji domain of the KDM5/JARID1 family of histone H3 lysine 4 demethylases. J Biol Chem.

[34] Kristensen LH, Nielsen AL, Helgstrand C, Lees M, Cloos P, Kastrup JS (2012). Studies of H3K4me3 demethylation by KDM5B/Jarid1B/PLU1 reveals strong substrate recognition in vitro and identifies 2,4-pyridine-dicarboxylic acid as an in vitro and in cell inhibitor. FEBS J.

[35] Williams-Ashman HG, Seidenfeld J, Galletti P (1982). Trends in the biochemical pharmacology of 5′-deoxy-5′-methylthioadenosine. Biochem Pharm.

[36] Hao F (2021). Systemic profiling of KDM5 subfamily signature in non-small-cell lung cancer. Int J Gen Med.

[37] Bueno MTD, Richard S (2013). SUMOylation negatively modulates target gene occupancy of the KDM5B, a histone lysine demethylase. Epigenetics.

[38] Tasaki T, Sriram SM, Park KS, Kwon YT (2012). The N-end rule pathway. Annu Rev Biochem.

[39] Zhou B, Zhu Y, Xu W, Zhou Q, Tan L, Zhu L (2021). Hypoxia stimulates SUMOylation-dependent stabilization of KDM5B. Front Cell Dev Biol.

[40] Lu W, Liu S, Li B, Xie Y, Adhiambo C, Yang Q (2014). SKP2 inactivation suppresses prostate tumorigenesis by mediating JARID1B ubiquitination. Oncotarget.

[41] Liu Y, Zhou Y (2022). Circ_0087960 stabilizes KDM5B by reducing SKP2 mediated ubiquitination degradation and promotes osteogenic differentiation in periodontal ligament stem cells. Regen Ther.

[42] Dorosz J, Kristensen LH, Aduri NG, Mirza O, Lousen R, Bucciarelli S (2019). Molecular architecture of the Jumonji C family histone demethylase KDM5B. Sci Rep.

[43] Chen Z, Zang J, Whetstine J, Hong X, Davrazou F, Kutateladze TG (2006). Structural insights into histone demethylation by JMJD2 family members. Cell.

[44] Jamshidi S, Catchpole S, Chen J, So CWE, Burchell J, Rahman KM (2021). KDM5B protein expressed in viable and fertile ΔARID mice exhibit no demethylase activity. Int J Oncol.

[45] Catchpole S, Spencer-Dene B, Hall D, Santangelo S, Rosewell I, Guenatri M (2011). PLU-1/JARID1B/KDM5B is required for embryonic survival and contributes to cell proliferation in the mammary gland and in ER+ breast cancer cells. Int J Oncol.

[46] DiTacchio L, Le HD, Vollmers C, Hatori M, Witcher M, Secombe J (2011). Histone lysine demethylase JARID1a activates CLOCK-BMAL1 and influences the circadian clock. Science.

[47] Liu X, Secombe J (2015). The histone demethylase KDM5 activates gene expression by recognizing chromatin context through its PHD reader motif. Cell Rep.

[48] Gaillard S, Charasson V, Ribeyre C, Salifou K, Pillaire M-J, Hoffmann J-S (2021). KDM5A and KDM5B histone-demethylases contribute to HU-induced replication stress response and tolerance. Biol Open.

[49] Jorgovanovic D, Song M, Wang L, Zhang Y (2020). Roles of IFN-γ in tumor progression and regression: a review. Biomark Res.

[50] Fragale A, Romagnoli G, Licursi V, Buoncervello M, Del Vecchio G, Giuliani C (2017). Antitumor effects of epidrug/IFNα combination driven by modulated gene signatures in both colorectal cancer and dendritic cells. Cancer Immunol Res.

[51] Hino M, Doihara H, Kobayashi K, Aoe M, Shimizu N (2003). Caveolin-1 as tumor suppressor gene in breast cancer. Surg Today.

[52] Xue S, Lam YM, He Z, Zheng Y, Li L, Zhang Y (2020). Histone lysine demethylase KDM5B maintains chronic myeloid leukemia via multiple epigenetic actions. Exp Hematol.

[53] Vizcaíno JA, Deutsch EW, Wang R, Csordas A, Reisinger F, Ríos D (2014). ProteomeXchange provides globally coordinated proteomics data submission and dissemination. Nat Biotechnol.

