# Supplementary material for: A truncated and catalytically inactive isoform of KDM5B histone demethylase accumulates in breast cancer cells and regulates H3K4 tri-methylation and gene expression
Source: Cancer Gene Ther. 2023 Jan 26;30(6):822–32. doi: 10.1038/s41417-022-00584-w (PMC10281864; doi:10.1038/s41417-022-00584-w)
Supplement: Supplementary file 1 — Supplementary Methods [file 41417_2022_584_MOESM1_ESM.docx]

# SUPPLEMENTARY INFORMATION FOR

**A truncated and catalytically inactive isoform of KDM5B histone demethylase accumulates in breast cancer cells and regulates H3K4 tri-methylation and gene expression**

Elena Di Nisio, Valerio Licursi, Cecilia Mannironi, Valentina Buglioni, Alessandro Paiardini, Giulia Robusti, Roberta Noberini, Tiziana Bonaldi, Rodolfo Negri^*^

*To whom correspondence should be addressed. E-mail: rodolfo.negri@uniroma1.it

# SUPPLEMENTARY METHODS

**Plasmids**

The KDM5B-NTT isoform overexpression plasmid (KDM5B_OHu64359D_pcDNA3.1+/C-(K)-DYK, Cat. OHu64359D), the empty vector (pcDNA3.1(+)-C-DYK) as a negative control, and the GFP plasmid (pcDNA3.1(+)-C-eGFP) as transfection control were purchased from GenScript (US) and grown in DH5α competent cells (ThermoFisher Scientific, US). Plasmids were extracted using the Qiagen plasmid MIDI kit (Qiagen, US).

**Cell Transfection**

MCF7 and MDA-MB-231 cells were cultured in high-glucose DMEM with 1% L-Glut and 10% FBS without P/S at 37°C in 5% CO_2_. 30 x10^3^ cells/cm^2^ and MCF7 cells and 25 x10^3^ cells/cm^2^ of MDA-MB-231 cells were seeded the day before transfection. Transfection using LIPO3000 (ThermoScientific, US) and Opti-MEM I Reduced Serum Medium (Gibco, ThermoFisher Scientific, US) was performed in 60-mm plates, using 9 μg of NTT overexpression plasmid or empty plasmids according to the manufacturer’s protocol, and cell lysates were prepared 24 and 48 hours after plasmid transfection for subsequent analysis.

**RNA extraction, RT-PCR and RT-qPCR**

Total RNA extraction was performed using the RNeasy Mini kit (Qiagen, US) or miRNeasy Mini kit with DNase on column digestion (Qiagen, US) according to the manufacturer’s protocol. After RNA extraction, RNA quality was tested by semi-denaturing gel electrophoresis using NorthernMax Formaldehyde Load Dye RNA (Ambion-ThermoFisher Scientific, US). Reverse transcription (RT) of mRNAs was performed using the SensiFAST cDNA synthesis kit, according to the manufacturer’s protocol (Meridian, US). PCR amplification was performed using DreamTaq Polymerase according to the manufacturer’s protocol guidelines (ThermoFisher Scientific, US). RT-qPCR was performed using a SensiFAST SYBR Hi-ROX Kit ((Meridian, US). PrimerBlast and Primer3 software were used for primer design. The primers used for RT-PCR and RT-qPCR are listed in Supplementary Table S1. RT-PCR was run as follows: 95 °C for 3 min, 95 °C for 30 s, 55 °C for 30 s, and 72 °C for 1 min for 35 cycles, with a final elongation at 72°C for 7 min. RT-qPCR was run in a two-step cycle at 95°C for 2 min, then 95 °C for 5 s, and 60 °C for 30 s for 40 cycles. Relative quantification of gene expression was conducted using the Applied Biosystems Step One Plus PCR System and data analysis was performed using the comparative ΔΔCt method. Actin was used as an endogenous reference gene.

**Protein structure analysis**

The crystal structure of the catalytic domain of PLU-1 in complex with N-oxalylglycine was retrieved from Protein Data Bank (www.rcsb.org; PDB: 5A1F; (1,2). KDM5B-NTT (residues 1-631) and the first 25 residues that were missing in Plu1 were *ab initio* modeled using the official release of the neural network implemented in AlphaFold2 (version 2.0.1; (3). PFAM (4) was used to map the positions of the JmjN, ARID, PHD, JmjC and zf-C5HC2 domains on the three-dimensional structures. The ELM server was used to identify potential degradation signals on the sequences of PLU1 and KDM5B-NTT (5). Protein sequences and structures analysis was carried out using PyMod 3 (6).

**Mass spectrometry (MS) analysis**

Histones were enriched from 1x10^6^ breast cancer cells (MDA-MB-231 and MCF7) in 6 biological replicates for each condition (NT, E, and KDM5B-NTT), as previously described (7). Prior to digestion, approximately 3 µg of histone octamer were mixed with an equal amount of heavy-isotope labelled histones, which were used as an internal standard for quantification (8), and were separated on a 17% SDS-PAGE gel. Histone bands were excised, chemically acylated with propionic anhydride and in-gel digested with trypsin, followed by peptide N-terminal derivatization with phenyl isocyanate (PIC) (9). Peptide mixtures were separated by reversed-phase liquid chromatography (RP-LC) on an EASY-Spray column (ThermoFisher Scientific, US), 25-cm long (inner diameter 75 µm, PepMap C18, 2 µm particles), which was connected online to a Q Exactive Plus instrument (Thermo Fisher Scientific) through an EASY-Spray™ Ion Source (ThermoFisher Scientific, US)(9).

The differentially modified peptides containing H3K4 were quantified manually using QualBrowser version 2.0.7 (ThermoFisher Scientific, US), as previously described (10). For each histone modified peptide, the light (sample) and heavy (internal standard) percentage relative abundances (%RAs) were estimated by dividing the area under the curve (AUC) of each modified peptide for the sum of the areas corresponding to all the observed forms of that peptide. Light/Heavy (L/H) ratios of %RA were then calculated (Table S2). The mass spectrometry proteomics data have been deposited to the ProteomeXchange Consortium (11) via the PRIDE partner repository with the dataset identifier PXD033337. Data display and statistical analysis were carried out using GraphPad Prism 9.3.1 (GraphPad). Changes in single histone modifications among groups were analyzed by one-way ANOVA, followed by Tukey's multiple comparisons test performed on log2 transformed L/H ratios.

**RNA-Sequencing**

Data for estimation of exon-1 and exon-6 containing transcripts of KDM5B are taken from the CCLE repository for tumour cell lines related data and from TCGA BRCA project for patients affected by breast cancer related data.

For RNA-Sequencing (RNA-Seq), total RNA was extracted from purified from cell pellets of control and KDM5B-NTT-overexpressing cultures using Qiagen RNeasy kits. RNA-Seq libraries from total RNA (1 μg) from each sample were prepared using the Illumina TruSeq Stranded mRNA Kit (Illumina, San Diego, CA) according to manufacturer’s instructions. The amplified fragmented cDNA of 300 bp in size were sequenced in paired-end mode using a NovaSeq6000 (Illumina) sequencer with a read length of 150 bp. Processing raw data for both format conversion and de-multiplexing were performed by Bcl2Fastq version 2.20 of the Illumina pipeline. Sequence reads quality was evaluated using *FastQC* (version 0.11.8, Babraham Institute Cambridge, UK) tool then adapter sequences were masked with Cutadapt version 1.11 from raw fastq data using the following parameters: *--anywhere --overlap 5 --times 2 --minimum-length 35 --mask-adapter.* Then reads were mapped to the mouse Ensembl GRCh38 build reference genome using *HISAT2* version 2.2.0 using Gene annotations according to the Ensembl database. Gene-level quantification was performed using *salmon* version 1.8.0 (12).

**Differential gene expression analysis**

To identify differentially expressed genes (DEGs) data was filtered to remove from the analysis the genes having < 10 counts per million across all replicates for each comparison.

DEGs were assessed with a comparison of MCF7 and MDA-MB-231 cell lines with constitutive expression of KDM5B-NTT over control cell lines, using a Wald’s test with a false discovery rate threshold of (FDR) < 0.1.

The data normalization and differential analysis for gene expression were performed using Bioconductor (13,14) R package *DESeq2* version 1.34 (15) accounting for the presence of batch effects. The figures were obtained using the R environment with base plot functions and from package *ggplot2* version 3.3.6. Volcano plots were created using Bioconductor R package *EnhancedVolcano* version 1.12.0.

**Gene set enrichment analysis (GSEA)**

Broad Institute’s Gene Set Enrichment Analysis (GSEA) (16) method was used to assess the enrichment of the ranked list of DEGs of the KDM5B-NTT unique gene expression signatures of MCF7 and MDA-MB-231 samples versus the curated “Hallmark” gene set collections from the BROAD molecular signature database (MSigDb version 7.4.1). The human version of the MSigDb “Hallmark” gene set collections were obtained from the *msigdbr* R package version 7.4.1.

This ES of GSEA is calculated by walking down the ranked list of genes, increasing a running-sum statistic when a gene is in the gene set and decreasing it when it is not. A normalized enrichment score (NES) is also calculated by GSEA in which differences in pathway size (i.e., gene set size) are considered, allowing for comparisons between pathways within the analysis. The enrichments were considered significant when FDR < 0.01.

**Transcriptome of MCF7 cells treated with epigenetic drugs**

MCF7 cells were treated with 60 μM 5′-deoxy-5′-methylthioadenosine (MTA) or with 300 μM 2,4-pyridine-dicarboxylic acid (PDCA) and 0.6% DMSO for negative controls. After 24h cells were harvested, and total RNA purified. The transcriptomes of MTA or PDCA treated cells were compared with DMSO treated cells.

**Cycloheximide assay and proteasome inhibitor**

MCF7 and MDA-MB-231 cells were seeded in 6-well plates at densities of 30 x10^3^ cells/well and 25 x10^3^ cells/well respectively. After 24h from the cell seeding, cells were treated with cycloheximide (CHX, 50 μg/ml) (Sigma-Aldrich, DE), using DMSO as a mock negative control, or CHX (50 μg/ml) and (R)-MG132 (10μM) (Sigma-Aldrich, DE). RIPA cell lysates were prepared every two hours of treatment.

**Cell Proliferation Assay**

Cell proliferation assay was performed with Cell Counting Kit-8 (Sigma-Aldrich), according to the manufacturer’s instructions. MCF7 cells were seeded in a 96-well plate at a density of 10^4^cells/well the day before transfection. The transfection was performed with KDM5B-NTT over-expression or empty vectors as described in the ‘Cell Transfection’ paragraph. At indicated time of cells growth a volume of 10 μL of CCK-8 solution was added to each well and plate incubated for 3 hours at 37°C in a 5% CO_2_ atmosphere. The absorbance at 450 nm was read using a Clariostar microplate reader (BMG Labtech).

**Flow-cytometry**

Flow-cytometry analysis of DNA content was performed using an EPICS xl flow-cytometer (Beckman-Coulter). At the indicated times from transfection, MCF7 cells were trypsinized, pelleted, washed with PBS and, finally, resuspended in PBS containing 1% Triton- X-100 and 40 μg/mL propidium iodide (Sigma-Aldrich). Samples were incubated for 20 min at 37°C and then analyzed, acquiring 10 000 events for each sample. Acquired data were analyzed using the WinMDI software by Joe Trotter, available at http:// facs.scripps.edu.

**REFERENCES**

1. Johansson C, Velupillai S, Tumber A, Szykowska A, Hookway ES, Nowak RP, et al. Structural analysis of human KDM5B guides histone demethylase inhibitor development. Nat Chem Biol. 2016;12:539–45.

2. Berman HM, Westbrook J, Feng Z, Gilliland G, Bhat TN, Weissig H, et al. The Protein Data Bank. Nucleic Acids Res. 2000;28:235–42.

3. Jumper J, Evans R, Pritzel A, Green T, Figurnov M, Ronneberger O, et al. Highly accurate protein structure prediction with AlphaFold. Nature. 2021;596:583–9.

4. Mistry J, Chuguransky S, Williams L, Qureshi M, Salazar GA, Sonnhammer ELL, et al. Pfam: The protein families database in 2021. Nucleic Acids Res. 2021;49:D412–9.

5. Puntervoll P, Linding R, Gemünd C, Chabanis-Davidson S, Mattingsdal M, Cameron S, et al. ELM server: A new resource for investigating short functional sites in modular eukaryotic proteins. Nucleic Acids Res. 2003;31:3625–30.

6. Janson G, Paiardini A. PyMod 3: a complete suite for structural bioinformatics in PyMOL. Bioinformatics. 2021;37:1471–2.

7. Noberini R, Restellini C, Savoia EO, Bonaldi T. Enrichment of histones from patient samples for mass spectrometry-based analysis of post-translational modifications. Methods. 2020;184:19–28.

8. Noberini R, Bonaldi T. A Super-SILAC Strategy for the Accurate and Multiplexed Profiling of Histone Posttranslational Modifications. Methods Enzymol. 2017;586:311–32.

9. Noberini R, Savoia EO, Brandini S, Greco F, Marra F, Bertalot G, et al. Spatial epi-proteomics enabled by histone post-translational modification analysis from low-abundance clinical samples. Clin Epigenetics. 2021;13:145.

10. Noberini R, Osti D, Miccolo C, Richichi C, Lupia M, Corleone G, et al. Extensive and systematic rewiring of histone post-translational modifications in cancer model systems. Nucleic Acids Res. 2018;46:3817–32.

11. Vizcaíno JA, Deutsch EW, Wang R, Csordas A, Reisinger F, Ríos D, et al. ProteomeXchange provides globally coordinated proteomics data submission and dissemination. Nat Biotechnol. 2014;32:223–6.

12. Patro R, Duggal G, Love MI, Irizarry RA, Kingsford C. Salmon provides fast and bias-aware quantification of transcript expression. Nat Methods 2017;14, 417–419.

13. Gentleman RC, Carey VJ, Bates DM, Bolstad B, Dettling M, Dudoit S, Ellis B, et al. Bioconductor: open software development for computational biology and bioinformatics. Genome Biol 2004;5, R80.

14. Huber W, Carey VJ, Gentleman R, Anders S, Carlson M, Carvalho BS, et al. Orchestrating high-throughput genomic analysis with Bioconductor. Nat. Methods 2015;12, 115–121.

15. Love MI, Huber W, Anders S. Moderated estimation of fold change and dispersion for RNA-seq data with DESeq2. Genome Biology 2014;15, 550.

16. Subramanian A, Tamayo P, Mootha VK, Mukherjee S, Ebert BL, Gillette MA, et al. Gene set enrichment analysis: a knowledge-based approach for interpreting genome-wide expression profiles. Proc Natl Acad Sci U S A. 2005;102(43):15545-50.
