# Supplementary material for: A truncated and catalytically inactive isoform of KDM5B histone demethylase accumulates in breast cancer cells and regulates H3K4 tri-methylation and gene expression
Source: Cancer Gene Ther. 2023 Jan 26;30(6):822–32. doi: 10.1038/s41417-022-00584-w (PMC10281864; doi:10.1038/s41417-022-00584-w)
Supplement: Supplementary file 2 — Supplementary Tables [file 41417_2022_584_MOESM2_ESM.docx]

**Table S1. Primers sequences**

| **RT-PCR Primers** | **Primer Sequences** |
| --- | --- |
| NM Fw | 5'-CCAAGATGGGGTTTGCTCCT-3' |
| NM Rv | 5'-CAGACATACAGGTCCACAGCA-3' |
| **RT-qPCR Primers** | **Primer Sequences** |
| Actin Fw | 5’-TCGTGCGTGACATTAAGGAG-3’ |
| Actin Rv | 5’-AGGAAGGAAGGCTGGAAGAG-3’ |
| ALL KDM5B Fw | 5’-TGCTTGATCCCACCTCTCCA-3’ |
| ALL KDM5B Rv | 5’-AACGCATCTGCCATTTCCCC-3’ |
| KDM5B Exon-6 Fw | 5’-CGAGCAAAACGCATGAGAGCA-3’ |
| KDM5B Exon-6 Rv | 5’-AGGCAGAAGAATTGCTGGAATCTA-3’ |
| **5’RACE Primers** | **Primer Sequences** |
| 5'RACE Outer Fw primer | 5'-GCTGATGGCGATGAATGAACACTG-3' |
| 5'RACE Inner Fw primer | 5'-CGCGGATCCGAACACTGCGTTTGCTGGCTTTGATG-3' |
| Reverse Ex-6 | 5'-AGGCAGAAGAATTGCTGGAATCTA-3' |
| Reverse 1 | 5'-AGGAGCAAACCCCATCTTGG-3' |
| Reverse 2 | 5'-TTTACACGAGTTTGGGCCTCC-3' |

# Table S2. MS-based quantification of H3K4 methylations (L/H ratios)

| MCF7 | NT | | | | | | E | | | | | | NTT | | | | | |
| --- | --- | --- | --- | --- | --- | --- | --- | --- | --- | --- | --- | --- | --- | --- | --- | --- | --- | --- |
|  | R#1 | R#2 | R#3 | R#4 | R#5 | R#6 | R#1 | R#2 | R#3 | R#4 | R#5 | R#6 | R#1 | R#2 | R#3 | R#4 | R#5 | R#6 |
| H3K4un | 0.99 | 0.99 | 0.99 | 0.98 | 0.99 | 1.00 | 1.00 | 0.99 | 0.99 | 0.98 | 1.01 | 1.00 | 0.99 | 0.99 | 0.99 | 0.96 | 0.99 | 1.00 |
| H3K4me1 | 1.16 | 1.21 | 1.13 | 1.11 | 1.04 | 1.02 | 1.14 | 1.17 | 1.14 | 1.06 | 1.02 | 1.07 | 1.13 | 1.21 | 1.15 | 1.15 | 1.02 | 1.02 |
| H3K4me2 | 0.84 | 0.87 | 0.87 | 0.93 | 0.92 | 0.88 | 0.79 | 0.94 | 0.90 | 0.85 | 0.78 | 0.77 | 0.89 | 0.90 | 0.92 | 0.98 | 0.88 | 0.86 |
| H3K4me3 | 0.85 | 0.98 | 0.88 | 0.93 | 1.18 | 1.16 | 0.60 | 1.13 | 0.78 | 1.17 | 0.77 | 0.53 | 1.03 | 1.17 | 1.19 | 1.26 | 1.28 | 1.19 |
| MDA-MB-231 | NT | | | | | | E | | | | | | NTT | | | | | |
|  | R#1 | R#2 | R#3 | R#4 | R#5 | R#6 | R#1 | R#2 | R#3 | R#4 | R#5 | R#6 | R#1 | R#2 | R#3 | R#4 | R#5 | R#6 |
| H3K4un | 1.00 | 1.01 | 1.01 | 1.01 | 1.04 | 0.98 | 1.00 | 1.00 | 1.00 | 1.02 | 0.98 | 0.98 | 1.00 | 1.00 | 1.00 | 1.01 | 0.99 | 0.99 |
| H3K4me1 | 0.88 | 0.87 | 0.85 | 0.80 | 0.78 | 0.92 | 0.95 | 0.96 | 0.96 | 0.87 | 0.96 | 0.92 | 0.94 | 0.90 | 0.97 | 0.86 | 0.95 | 1.00 |
| H3K4me2 | 1.31 | 1.29 | 1.20 | 1.12 | 1.12 | 1.38 | 1.32 | 1.20 | 1.26 | 1.22 | 1.29 | 1.31 | 1.26 | 1.29 | 1.26 | 1.23 | 1.36 | 1.26 |
| H3K4me3 | 1.26 | 1.05 | 1.16 | 1.24 | 1.20 | 1.46 | 1.28 | 1.25 | 1.29 | 0.97 | 1.34 | 1.06 | 1.17 | 1.20 | 1.19 | 1.17 | 1.21 | 1.20 |
