# Supplementary material for: A truncated and catalytically inactive isoform of KDM5B histone demethylase accumulates in breast cancer cells and regulates H3K4 tri-methylation and gene expression
Source: Cancer Gene Ther. 2023 Jan 26;30(6):822–32. doi: 10.1038/s41417-022-00584-w (PMC10281864; doi:10.1038/s41417-022-00584-w)
Supplement: Supplementary file 3 — Supplementary File S1 [file 41417_2022_584_MOESM3_ESM.docx]

# Supplementary File S1

#

# Alignment of 5’RACE PCR products using the PLU-1 transcript as a reference

# (First and second ATG, Anchor primer sequence, and transcriptional start site of NTT transcript are highlighted)

PCR2_oligoRev ------------------------------------------------------------ 0

PCR1_oligoFor ------------------------------------------------------------ 0

NM_006618.5 GTACAACTCGGACTTGCTGTTGCTCGAGCCGCGTCTGCACGGGTCTCGGACCGAGCGGAG 60

PCR1_oligoRev ------------------------------------------------------------ 0

PCR2_oligoFor ------------------------------------------------------------ 0

PCR2_oligoRev ------------------------------------------------------------ 0

PCR1_oligoFor ------------------------------------------------------------ 0

NM_006618.5 CTCGCAGCCTCGGTCCCGGAGCCCACCTTCGCCTCGCCCTTGCCCAGCCTGCGGTG**ATG**G 120

PCR1_oligoRev ------------------------------------------------------------ 0

PCR2_oligoFor ------------------------------------------------------------ 0

PCR2_oligoRev ------------------------------------------------------------ 0

PCR1_oligoFor ------------------------------------------------------------ 0

NM_006618.5 AGGCGGCCACCACACTGCACCCAGGCCCGCGCCCGGCGCTGCCCCTCGGGGGCCCGGGCC 180

PCR1_oligoRev ------------------------------------------------------------ 0

PCR2_oligoFor ------------------------------------------------------------ 0

PCR2_oligoRev ---------TCGCGGATCCGAACACTGCGTTTGCTGGCTTC**G**AACCCAGCTGGGAAGAGT 51

PCR1_oligoFor -----------------------------------CCCTTCGATCAGCTGTTCCAGAGTC 25

NM_006618.5 CGCTGGGCGAGTTCCTGCCTCCACCCGAGTGCCCGGTCTTCGAACCCAGCTGGGAAGAGT 240

PCR1_oligoRev ----------CGCGGATCCGAACACTGCGTTTGCTGGCTTC**G**AACCCAGCTGGGAAGAGT 50

PCR2_oligoFor -------------------------------------------GGCTGACAGCTGGGAGA 17

PCR2_oligoRev TCGCGGACCCCTTCGCTTTCATCCACAAGATCCGGCCCATAGCCGAGCAGACTGGCATCT 111

PCR1_oligoFor GCGAGATGCTTCGACTTCTCATCCACAAGATCCGGCCCATAGCCGAGCAGACTGGCATCT 85

NM_006618.5 TCGCGGACCCCTTCGCTTTCATCCACAAGATCCGGCCCATAGCCGAGCAGACTGGCATCT 300

PCR1_oligoRev TCGCGGACCCCTTCGCTTTCATCCACAAGATCCGGCCCATAGCCGAGCAGACTGGCATCT 110

PCR2_oligoFor GTCGCGGACCCTTCGCTTTCATCCACAAGATCCGGCCCATAGCCGAGCAGACTGGCATCT 77

* * ******************************************

PCR2_oligoRev GTAAGGTGCGGCCGCCGCCGGATTGGCAGCCACCATTTGCATGTGATGTTGAAAACTCAT 171

PCR1_oligoFor GTAAGGTGCGGCCGCCGCCGGATTGGCAGCCACCATTTGCATGTGATGTTGATAAACTTC 145

NM_006618.5 GTAAGGTGCGGCCGCCGCCGGATTGGCAGCCACCATTTGCATGTGATGTTGATAAACTTC 360

PCR1_oligoRev GTAAGGTGCGGCCGCCGCCGGATTGGCAGCCACCATTTGCATGTGATGTTGATAAACTTC 170

PCR2_oligoFor GTAAGGTGCGGCCGCCGCCGGATTGGCAGCCACCATTTGCATGTGATGTTGATAAACTTC 137

**************************************************** **

PCR2_oligoRev TACGCCAAGACACAGCCCTATAG------------------------------------- 194

PCR1_oligoFor ATTTTACGCCACGTATCCAGAGACTGAATGAATTGGAGGCCCAAACTCGTGTAAAATTGA 205

NM_006618.5 ATTTTACGCCACGTATCCAGAGACTGAATGAATTGGAGGCCCAAACTCGTGTAAAATTGA 420

PCR1_oligoRev ATTTTACGCCACGTATCCAGAGACTGAATGAATTGGAGGCCCAAACTCGTGTAAAATTGA 230

PCR2_oligoFor ATTTTACGCCACGTATCCAGAGACTGAATGAATTGGAGGCCCAAACTCGTGTAAAA---- 193

PCR2_oligoRev ------------------------------------------------------------ 194

PCR1_oligoFor ATTTCTTGGACCAGATTGCAAAGTACTGGGAGTTACAGGGAAGTACTCTGAAAATTCCAC 265

NM_006618.5 ATTTCTTGGACCAGATTGCAAAGTACTGGGAGTTACAGGGAAGTACTCTGAAAATTCCAC 480

PCR1_oligoRev ATTTCTTGGACCAGATTGCAAAGTACTGGGAGTTACAGGGAAGTACTCTGAAAATTCCAC 290

PCR2_oligoFor ------------------------------------------------------------ 193

PCR2_oligoRev ------------------------------------------------------------ 194

PCR1_oligoFor ATGTGGAGAGGAAGATCTTGGACTTATTTCAGCTTAATAAGTTAGTTGCAGAAGAAGGTG 325

NM_006618.5 ATGTGGAGAGGAAGATCTTGGACTTATTTCAGCTTAATAAGTTAGTTGCAGAAGAAGGTG 540

PCR1_oligoRev ATGTGGAGAGGAAGATCTTGGACTTATTTCAGCTTAATAAGTTAGTTGCAGAAGCAAGGT 350

PCR2_oligoFor ------------------------------------------------------------ 193

PCR2_oligoRev ------------------------------------------------------------ 194

PCR1_oligoFor GATTTGCAGT-TGTTTGCAAGGATAGAAAATGGACCAAAATTGCTACCAAG**ATG**GGGTTT 384

NM_006618.5 GATTTGCAGT-TGTTTGCAAGGATAGAAAATGGACCAAAATTGCTACCAAG**ATG**GGGTTT 599

PCR1_oligoRev GGATTTGTCAGTTGTTGCAAGGATATAGTAGCCTTCTAAAG------------------- 391

PCR2_oligoFor ------------------------------------------------------------ 193

PCR2_oligoRev ------------------------------------------------------------ 194

PCR1_oligoFor GCTCCTGA---------------------------------------------------- 392

NM_006618.5 GCTCCTGGCAAAGCAGTGGGCTCACATATCAGAGGGCATTATGAACGAATTCTCAACCCC 659

PCR1_oligoRev ------------------------------------------------------------ 391

PCR2_oligoFor ------------------------------------------------------------ 193
