# Supplementary material for: A truncated and catalytically inactive isoform of KDM5B histone demethylase accumulates in breast cancer cells and regulates H3K4 tri-methylation and gene expression
Source: Cancer Gene Ther. 2023 Jan 26;30(6):822–32. doi: 10.1038/s41417-022-00584-w (PMC10281864; doi:10.1038/s41417-022-00584-w)
Supplement: Supplementary file 4 — Supplementary Figures [file 41417_2022_584_MOESM4_ESM.pdf]

Figure S1

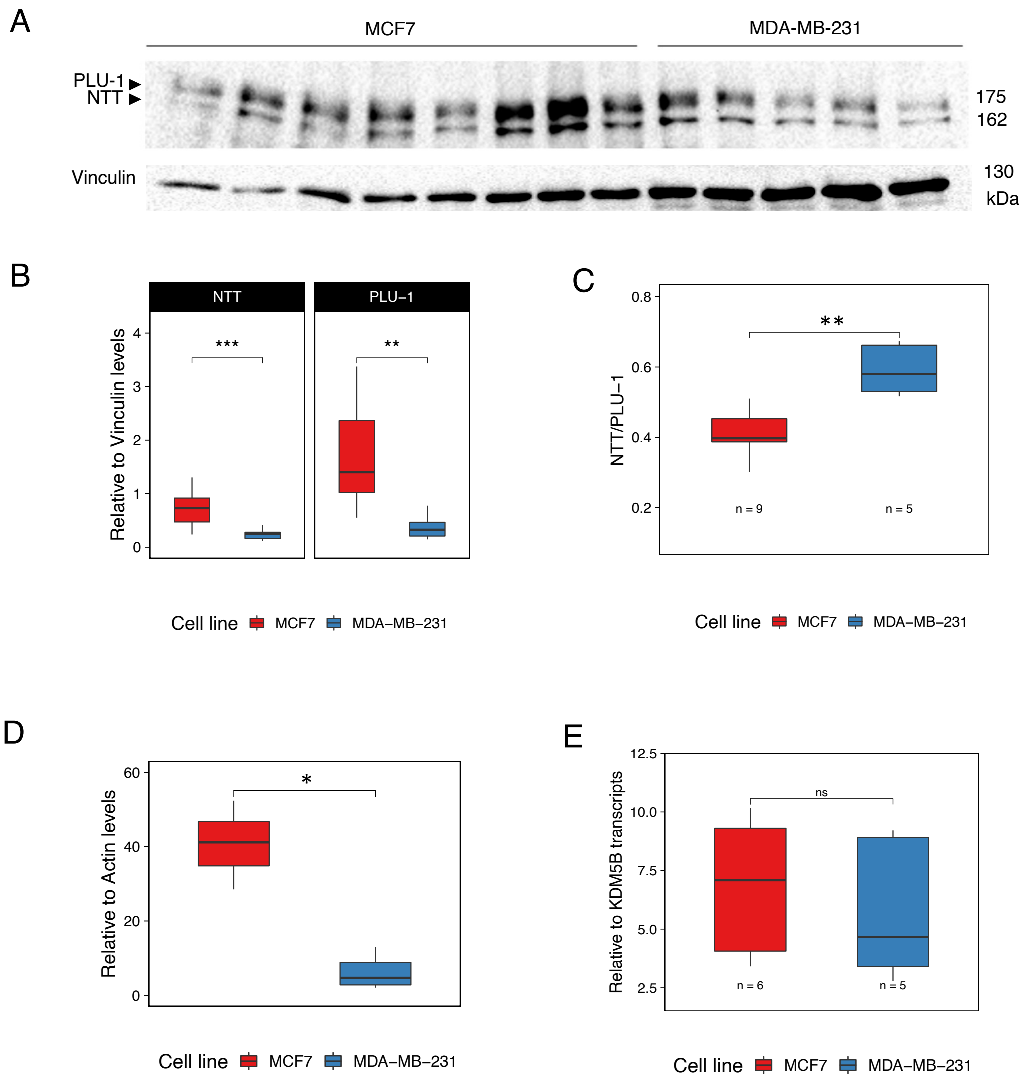

Figure S1. Expression of KDM5B protein isoforms and transcripts in MCF7 and MDA-MB-231 cells. **A**, Western blot using the anti-C-terminal Ab showing the KDM5B-PLU-1 (175KDa) and the KDM5B-NTT (162KDa) bands in several cultures of MCF7 and MDA-MB-231. **B**, A significative difference in the expression of these KDM5B isoforms relative to vinculin was observed in the two analyzed breast cancer cell lines; in particular, the data confirm that PLU-1 is more expressed in MCF7 rather that MDA-MB-231 and that the difference in the expression level of PLU-1 and NTT is grater in MCF7 cells; indeed, the expression level of PLU-1 and NTT is more similar in MDA-MB-231. **C**, Direct comparison with the data reported in the histogram Fig.1F. The NTT/PLU-1 ratio is higher in MDA-MB-231 versus MCF7, around 50-60% and 30-40% respectively. **D**, RT-qPCR shows that the total KDM5B transcripts' level in MCF7 is significantly higher compared to MDA-MB-231. **E,** Even though the relative expression of the NTT protein isoform over PLU-1 is significantly different in MCF7 and MDA-MB-231 (see panel C), the fraction of KDM5B transcripts including the exon-6 is similar in MCF7 and MDA-MB-231 cells.
ns: $p > 0.05$, * $p <= 0.05$, ** $p <= 0.01$, *** $p <= 0.001$

Figure S2

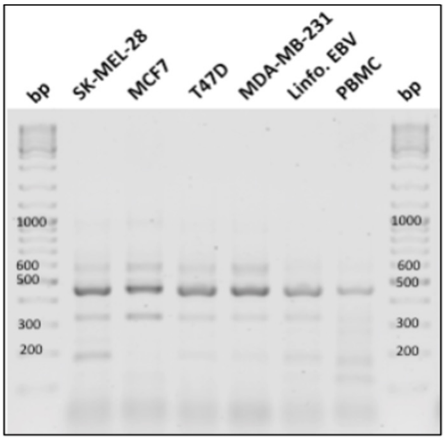

Fw Primer	Rv Primer	Amplicon Length (bp)	Transcript
NM Fw	NM Rv	574	RBP2-H1; X1; X2
		466	PLU-1; KDM5B-215
		331	KDM5B-240

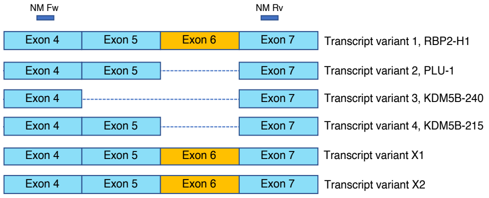

Figure S2. The RT-PCR with the NM primer pair (at right) produce all the expected amplicons, demonstrating the existence of different KDM5B splicing variants in breast cancer cell lines with a similar expression pattern to melanoma ones. Indeed, in SK-MEL-28, MCF7, T47D, and MDA-MB-231 there are KDM5B transcripts that include the exon-6 (RBP2H1/X1/X2, at 574 bp) and which are not present in the normal-control cell lines. The bands of 466 bp correspond to PLU-1 and/or KDM5B-215; the bands at 331 bp correspond to KDM5B-240.

Figure S3

A

PLU-1_nuclear localization signals: **I, aa 227–234**; **II, aa 295–302**; **III, aa 1103–1120**; **IV, aa 1400–1417**; **V, aa 1434–1440**. *From Lu et al. (1999)*

>(NP_006609.3) Lysine-specific demethylase 5B; PLU-1 isoform

```
MEAATTLHPGPRPALPLGGPGPLGEFLPPPECPVFEPSWEEFADPFAFIHKIRPIAEQTG  60
ICKVRPPPDWQPPFACDVDKLHFTPRIQRLNELEAQTRVKLNFLDQIAKYWELQGSTLKI  120
PHVERKILDLFQLNKLVAEEGGFAVVCKDRKWTKIATKMGFAPGKAVGSHIRGHYERILN  180
PYNLFLSGDSLRCLQKPNLTTDTKDKEYKPHDIPQRQSVQPSETCPPARRAKRMRAEAMN  240
IKIEPEETTEARTHNLRRRMGCPTPKCENEKEMKSSIKQEPIERKDYIVENEKEKPKSRS  300
KKATNAVDLYVCLLCGSGNDEDRLLLCDGCDDSYHTFCLIPPLHDVPKGDWRCPKCLAQE  360
CSKPQEAFGFEQAARDYTLRTFGEMADAFKSDYFNMPVHMVPTELVEKEFWRLVSTIEED  420
VTVEYGADIASKEFGSGFPVRDGKIKLSPEEEEYLDSGWNLNNMPVMEQSVLAHITADIC  480
GMKLPWLYVGMCFSSFCWHIEDHWSYSINYLHWGEPKTWYGVPGYAAEQLENVMKKLAPE  540
LFVSQPDLLHQLVTIMNPNTLMTHEVPVYRTNQCAGEFVITFPRAYHSGFNQGFNFAEAV  600
NFCTVDWLPLGRQCVEHYRLLHRYCVFSHDEMICKMASKADVLDVVVASTVQKDMAIMIE  660
DEKALRETVRKLGVIDSERMDFELLPDDERQCVKCKTTCFMSAISCSCKPGLLVCLHHVK  720
ELCSCPPYKYKLRYRYTLDDLYPMMNALKLRAESYNEWALNVNEALEAKINKKKSLVSFK  780
ALIEESEMKKFPDNDLLRHLRLVTQDAEKCASVAQQLLNGKRQTRYRSGGGKSQNQLTVN  840
ELRQFVTQLYALPCVLSQTPLLKDLLNRVEDFQQHSQKLLSEETPSAAELQDLLDVSFEF  900
DVELPQLAEMRIRLEQARWLEEVQQACLDPSSLTLDDMRRLIDLGVGLAPYSAVEKAMAR  960
LQELLTVSEHWDDKAKSLLKARPRHSLNSLATAVKEIEEIPAYLPNGAALKDSVQRARDW  1020
LQDVEGLQAGGRVPVLDTLIELVTRGRSIPVHLNSLPRLETLVAEVQAWKECAVNTFLTE  1080
NSPYSLLEVLCPRCDIGLLGLKRKQRKLKEPLPNGKKKSTKLESLSDLERALTESKETAS  1140
AMATLGEARLREMEALQSLRLANEGKLLSPLQDVDIKICLCQKAPAAPMIQCELCRDAFH  1200
TSCVAVPSISQGLRIWLCPHCRRSEKPPLEKILPLLASLQRIRVRLPEGDALRYMIERTV  1260
NWQHRAQQLLSSGNLKFVQDRVGSGLLYSRWQASAGQVSDTNKVSQPPGTTSFSLPDDWD  1320
NRTSYLHSPFSTGRSCIPLHGVSPEVNELLMEAQLLQVSLPEIQELYQTLLAKPSPAQQT  1380
DRSSPVRPSSEKNDCCRGKRDGINSLERKLKRRLEREGLSSERWERVKKMRTPKKKKIKL  1440
SHPKDMNNFKLERERSYELVRSAETHSLPSDTSYSEQEDSEDEDAICPAVSCLQPEGDEV  1500
DWVQCDGSCNQWFHQVCVGVSPEMAEKEDYICVRCTVKDAPSRK                  1544
```

>Lysine-specific demethylase 5B; NTT isoform

```
MGFAPGKAVGSHIRGHYERILNPYNLFLSGDSLRCLQKPNLTTDTKDKEYKPHDIPQRQS  60
VQPSETCPPARRAKRMRAERQSLAVLPRLECSGAILAHCNLRLLDSSNSSASASQAMNIK  120
IEPEETTEARTHNLRRRMGCPTPKCENEKEMKSSIKQEPIERKDYIVENEKEKPKSRSKK  180
ATNAVDLYVCLLCGSGNDEDRLLLCDGCDDSYHTFCLIPPLHDVPKGDWRCPKCLAQECS  240
KPQEAFGFEQAARDYTLRTFGEMADAFKSDYFNMPVHMVPTELVEKEFWRLVSTIEEDVT  300
VEYGADIASKEFGSGFPVRDGKIKLSPEEEEYLDSGWNLNNMPVMEQSVLAHITADICGM  360
KLPWLYVGMCFSSFCWHIEDHWSYSINYLHWGEPKTWYGVPGYAAEQLENVMKKLAPELF  420
VSQPDLLHQLVTIMNPNTLMTHEVPVYRTNQCAGEFVITFPRAYHSGFNQGFNFAEAVNF  480
CTVDWLPLGRQCVEHYRLLHRYCVFSHDEMICKMASKADVLDVVVASTVQKDMAIMIEDE  540
KALRETVRKLGVIDSERMDFELLPDDERQCVKCKTTCFMSAISCSCKPGLLVCLHHVKEL  600
CSCPPYKYKLRYRYTLDDLYPMMNALKLRAESYNEWALNVNEALEAKINKKKSLVSFKAL  660
IEESEMKKFPDNDLLRHLRLVTQDAEKCASVAQQLLNGKRQTRYRSGGGKSQNQLTVNEL  720
RQFVTQLYALPCVLSQTPLLKDLLNRVEDFQQHSQKLLSEETPSAAELQDLLDVSFEFDV  780
ELPQLAEMRIRLEQARWLEEVQQACLDPSSLTLDDMRRLIDLGVGLAPYSAVEKAMARLQ  840
ELLTVSEHWDDKAKSLLKARPRHSLNSLATAVKEIEEIPAYLPNGAALKDSVQRARDWLQ  900
DVEGLQAGGRVPVLDTLIELVTRGRSIPVHLNSLPRLETLVAEVQAWKECAVNTFLTENS  960
PYSLLEVLCPRCDIGLLGLKRKQRKLKEPLPNGKKKSTKLESLSDLERALTESKETASAM  1020
ATLGEARLREMEALQSLRLANEGKLLSPLQDVDIKICLCQKAPAAPMIQCELCRDAFHTS  1080
CVAVPSISQGLRIWLCPHCRRSEKPPLEKILPLLASLQRIRVRLPEGDALRYMIERTVNW  1140
QHRAQQLLSSGNLKFVQDRVGSGLLYSRWQASAGQVSDTNKVSQPPGTTSFSLPDDWDNR  1200
TSYLHSPFSTGRSCIPLHGVSPEVNELLMEAQLLQVSLPEIQELYQTLLAKPSPAQQTDR  1260
SSPVRPSSEKNDCCRGKRDGINSLERKLKRRLEREGLSSERWERVKKMRTPKKKKIKLSH  1320
PKDMNNFKLERERSYELVRSAETHSLPSDTSYSEQEDSEDEDAICPAVSCLQPEGDEVDW  1380
VQCDGSCNQWFHQVCVGVSPEMAEKEDYICVRCTVKDAPSRK                    1422
```

B

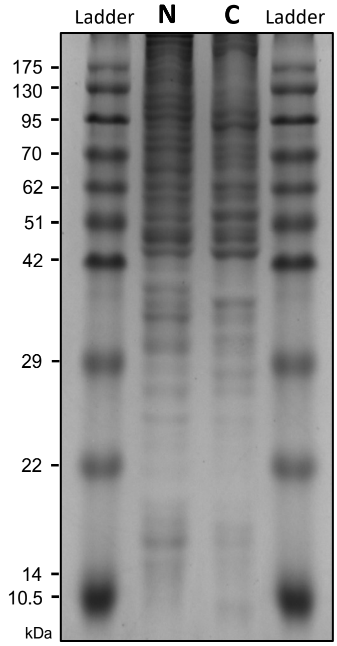

C

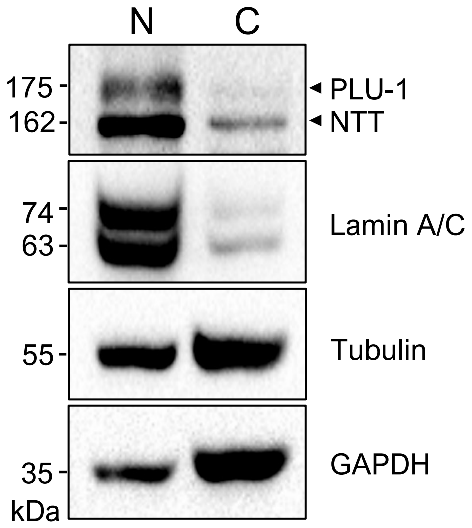

Figure S3. Analysis of NTT isoform localization. **A,** Nuclear Localization Signals (NLSs) of PLU-1 according to Lu et al. 1999 (14); all the NLSs are also in the NTT isoform suggesting that it can localize into the nucleus. **B,** Comassie blu staining after SDS-PAGE of nuclear (N) and cytoplasmic (C) fraction protein exctracts. The same total amount of protein extract was loaded for the nuclear and for the cytoplasmic fraction. **C,** Western blot with anti-C-term KDM5B, anti-LAM A/C, anti-Tubulin, anti-GAPDH in MDA-MB-231 cells of nuclear and cytoplasmic protein extracts confirms that also the NTT isoform localizes predominantly into the nucleus.

Figure S4

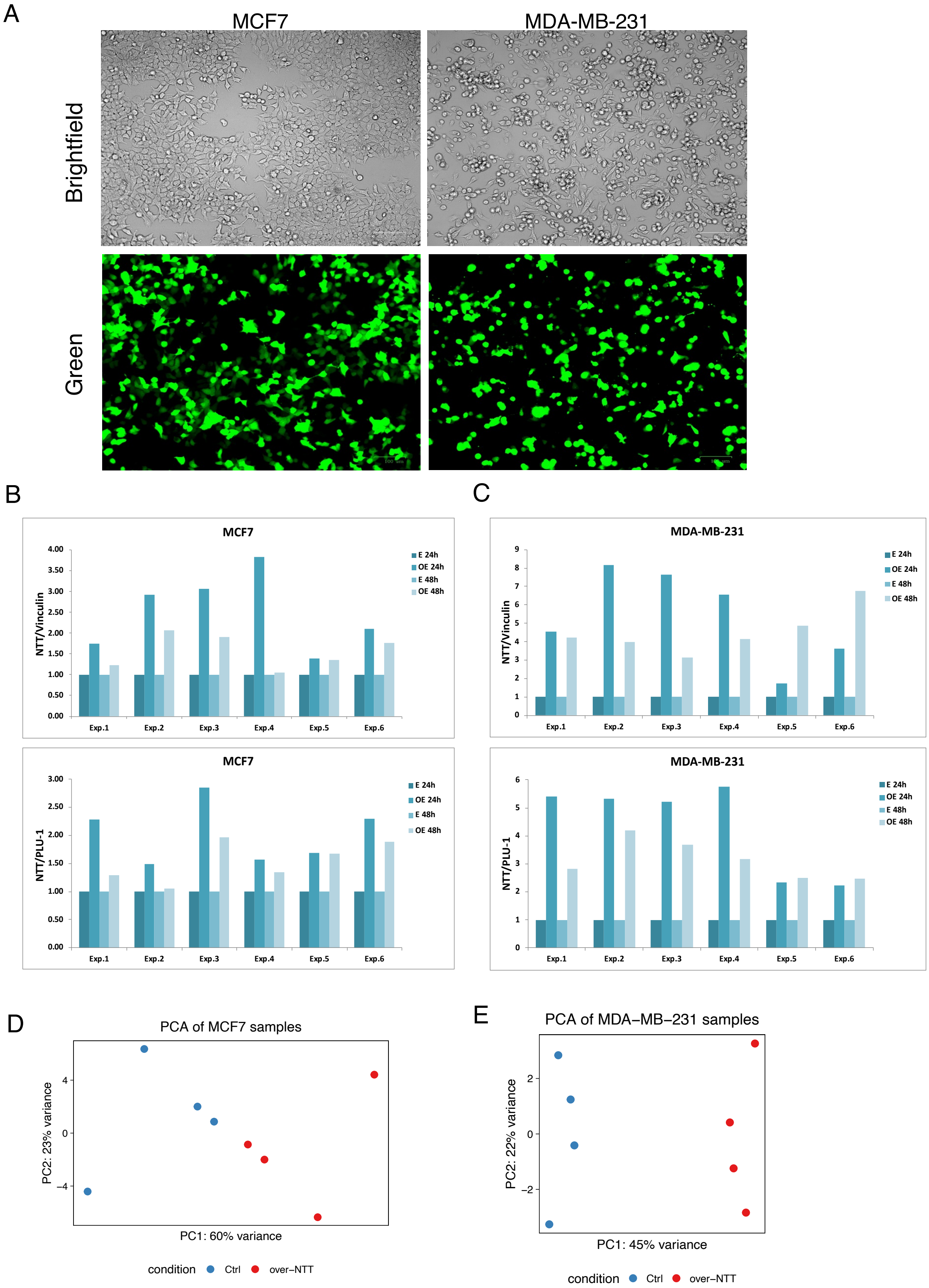

Figure S4. **A,** Post-transfection analysis of cells. Green fluorescence and brightfield images demonstrate about 60% and 50% transfection efficiency in MCF7 and MDA-MB-231. **B**, The overexpression of NTT compared to the control condition (E, Empty vector) was higher at 24 hours (OE 24h) rather than at 48 hours (OE 48h) from transfection in MCF7 cells; the quantification using vinculin as reference or the relative amount of NTT over PLU-1 is shown. **C**, The NTT protein was efficiently expressed also in MDA-MB-231, both at 24h and 48 h after transfection; over-expression quantification relative to Empty control conditions is shown both using vinculin as a calibrator and using the relative amount of NTT over PLU-1. **D**, Principal Component Analysis (PCA) after batch effect correction shows that gene expression profiles of MCF7 and MDA-MB-231. **E**, cells samples' replicates over-expressing NTT cluster differently from control replicates and most of the variance observed (explained by PC1) can be associated to the NTT over-expression.

Figure S5

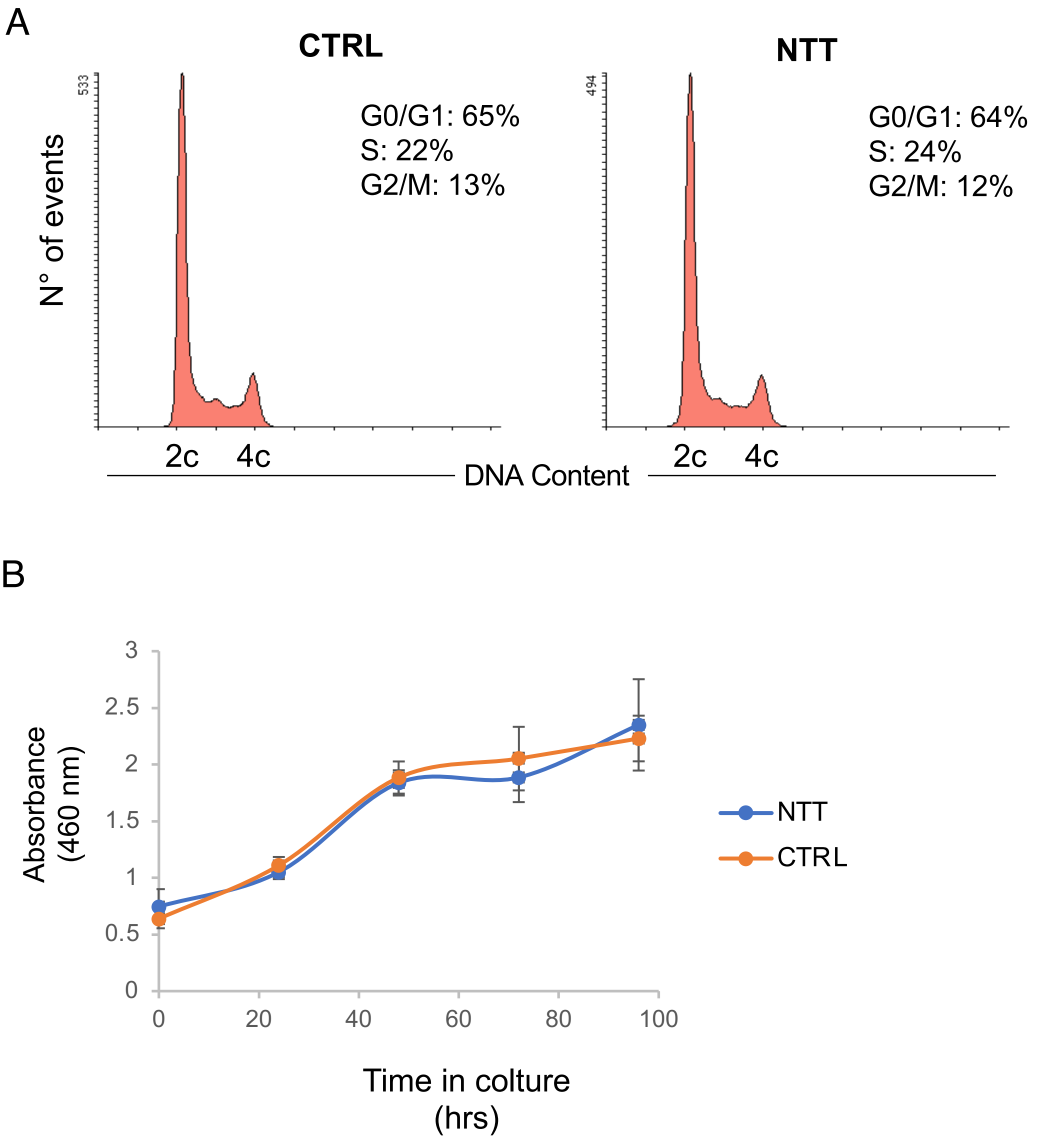

Figure S5. NTT over-expression in MCF7 cells does not affect cell cycle. **A,** Examples of cytofluorimetric patterns obtained from control cells (CTRL) and PLU-1-NTT over-expressing cells (NTT). Cells were transfected with empty and PLU-1-NTT vectors, respectively. Flow-cytometry analysis of DNA content was done at 24 hrs from transfection. Cell cycle phase distribution is reported as an inset in the figures. **B,** NTT over-expression in MCF7 cells does not affect cell proliferation. MCF7 cells were transfected with NTT over-expression vector or empty control vector. Cell proliferation were assayed by the WST-8 colorimetric assay. No significant differences were observed between control and NTT overexpressing cells. Each point represents the average of 8 biological replicates, vertical bars indicate standard deviation values.

Figure S6

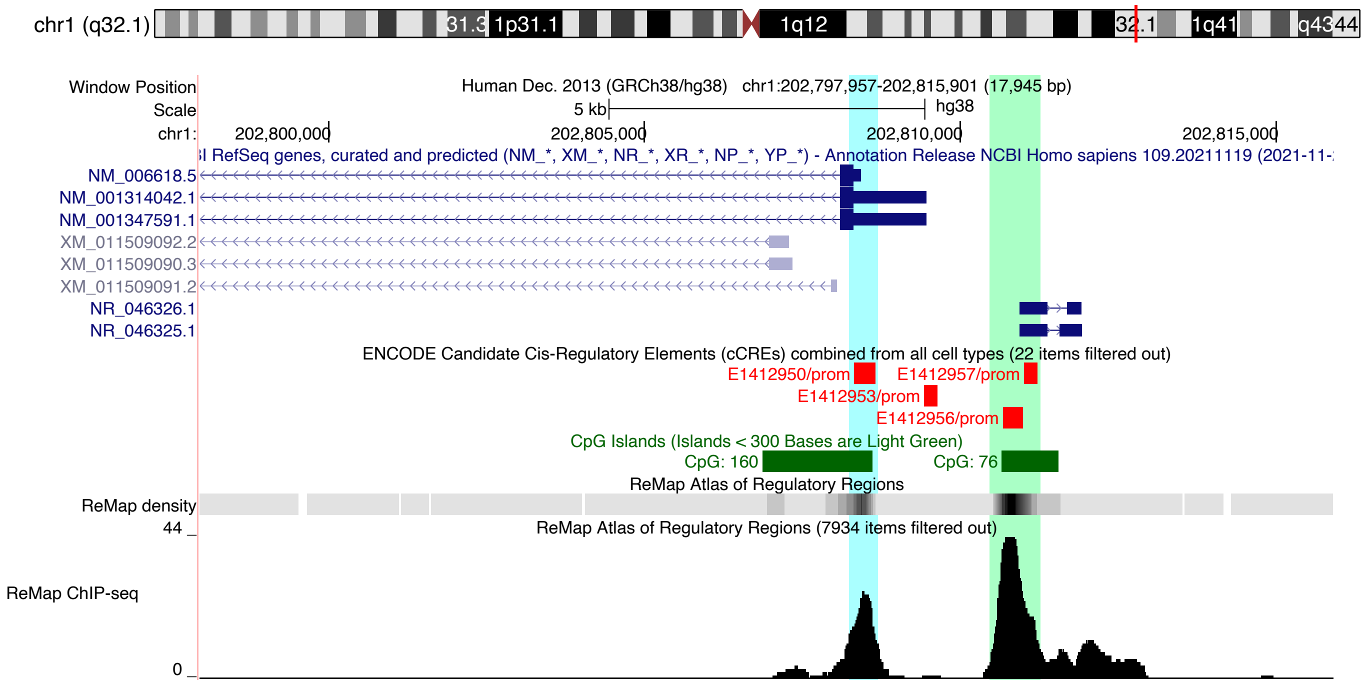

Figure S6. Promoter region of KDM5B gene using the UCSC genome browser. The figure shows the TSS region and the 5' end of KDM5B transcripts with curated (NM) and predicted (XM) RefSeq gene annotation (the last version of RefSeq available on UCSC genome browser is the 109.20211119 of 2021-11-23), followed by track with the ENCODE Registry of candidate cis-Regulatory Elements (cCREs) in the human genome. The red boxes of this track represent the cCREs with promoter-like signatures (cCRE-PLS) provided by the SCREEN (Search Candidate cis-Regulatory Elements) web tool. The green boxes represent prediction of two CpG islands. The last track shows the ReMap Atlas of regulatory regions, which consists of a large-scale integrative analysis of all Public ChIP-seq data for transcriptional regulators from GEO, ArrayExpress, and ENCODE. In particular here there are two ReMap subtracks with the first one showing the whole ChIP-seq dataset in "dense" mode while the second one showing the ChIP-seq dataset filtered for "breast" keyword in the biotype field of the dataset in "full" mode, respectively. These data clearly supports the existence of two promoter regions which are highlighted in light green and light blue. The figure was created with UCSC genome browser.

Figure S7

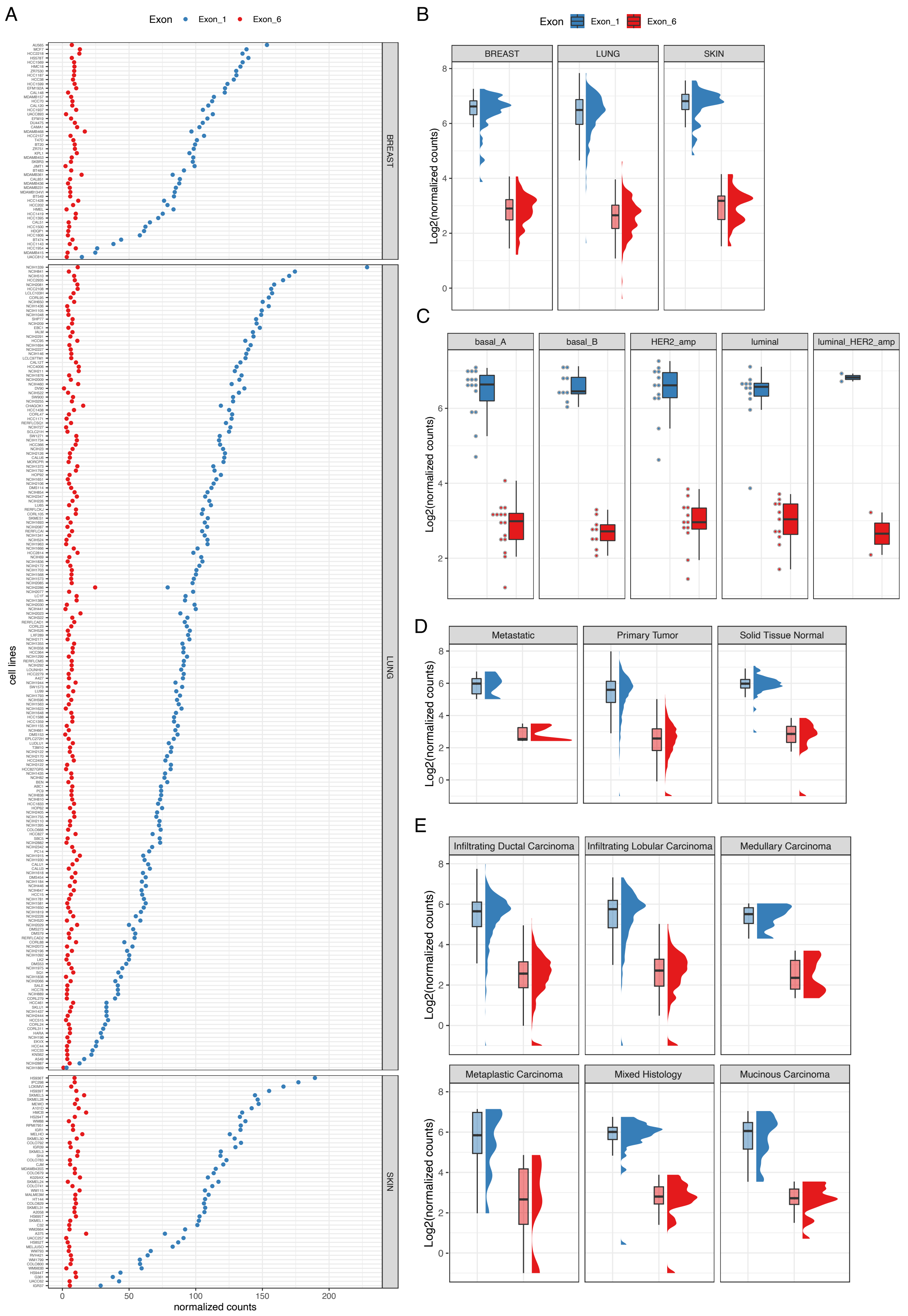

Figure S7. Large scale data analysis on publicly available data on KDM5B transcripts expression. **A,** Dot plots show the expression levels quantified by RNA-Seq of the KDM5B exon-1 and exon-6 for all the cell lines of Breast, Lung and Skin analyzed by the Cancer Cell Line Encyclopedia project (CCLE, Broad Institute, https://sites.broadinstitute.org/ccle/datasets). Data are reported in normalized count levels. **B,** Summaries of the data showed in (A) represented with boxplots and density curves. **C,** Boxplots showing the expression levels of the KDM5B exon-1 and exon-6 containing transcripts of Breast cancer cell lines of (A) grouped by tumor subtypes. **D,** Boxplots showing the expression levels of the KDM5B exon-1 and exon-6 containing transcripts in Breast Cancer samples of patients collected in TCGA project. The data are from 7 metastatic samples, 1127 primary tumors and 112 normal breast tissues samples. **E,** Summaries of the data showed in (D) stratified by histological breast cancer subtypes.

Figure S8

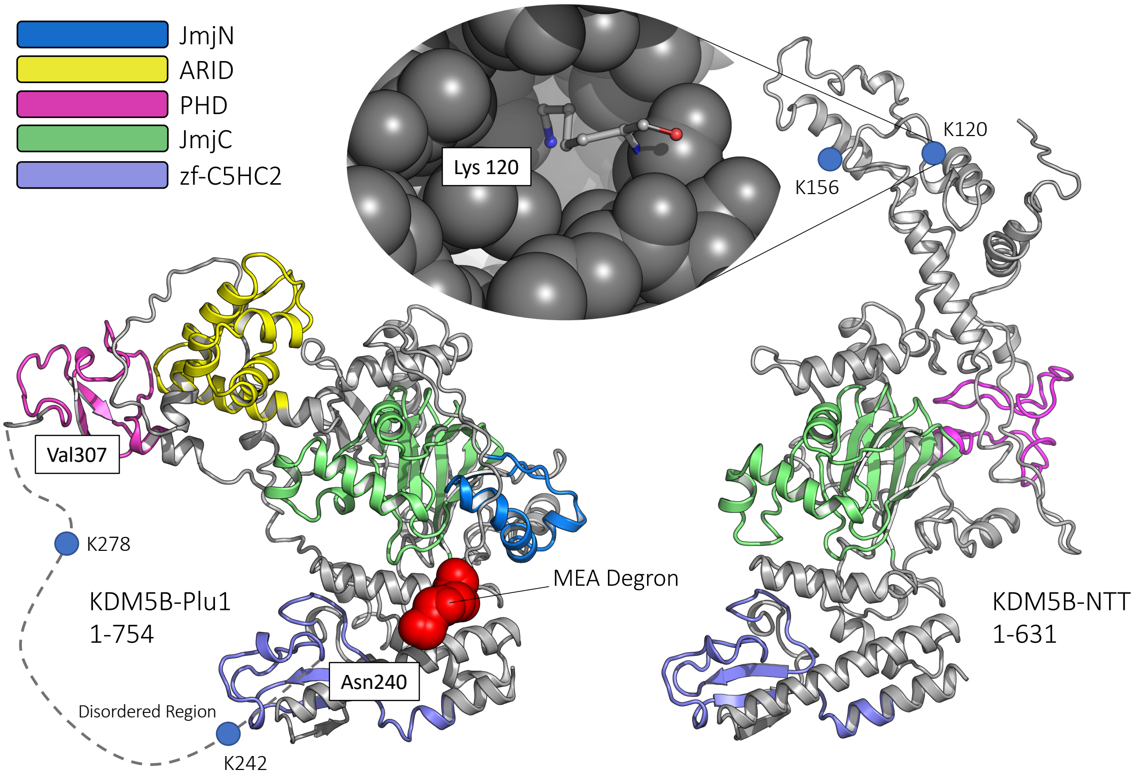

Figure S8. AlphaFold2 models of KDM5B-Plu1 (1-754; left) and the corresponding N-terminal region of KDM5B-NTT (1-631; right). The positions of the JmjN, ARID, PHD, JmjC and zf-C5HC2 domains were obtained by PFAM, mapped on the corresponding 3D-structures and colored according to the legend shown. The region 740-307, comprising the ubiquitinylated residues K242 and K278 (blue spheres) of KDM5B-Plu1, is predicted as intrinsically disordered and shown as dashed lines. The corresponding residues of NTT, K120 and K156, are predicted on a folded domain. In particular K120 is predicted as buried and not accessible for ubiquitinoylation. Sequence analysis with the ELM server indicates that Plu-1 has an N-terminal Degron which is missing in NTT ("MEA"). These structural differences may help partly explain the increased stability of NTT over Plu-1.
